# Assessing the disease burden of lower respiratory infections attributable to particulate matter pollution: trends from 1990 to 2021 and projections for 2022-2050

**DOI:** 10.3389/fcimb.2025.1660032

**Published:** 2025-10-23

**Authors:** Erman Wu, Tong Tang, Guohua Zhu, Chang Du, Dangmurenjiafu Geng

**Affiliations:** ^1^ Department of Neurosurgery, First Affiliated Hospital of Xinjiang Medical University, Urumqi, Xinjiang, China; ^2^ Department of Computer Science and Information Technologies, University of A Coruña, A Coruña, Spain; ^3^ School of Life Science, South China Normal University, Guangzhou, China

**Keywords:** global disease burden, particulate matter pollution, air pollution, lower respiratory infections, burden forecasting

## Abstract

**Background:**

Particulate matter pollution (PMP) remains a leading risk factor for lower respiratory infections (LRIs) globally. However, the evolving contributions of household air pollution (HAP) versus ambient particulate matter pollution (APMP) across different development stages remain poorly characterized, hindering targeted intervention strategies.

**Methods:**

Using Global Burden of Disease 2021 data from 204 countries (1990-2021), we analyzed age-standardized mortality rates (ASMR) and disability-adjusted life years (DALY) for PMP-attributable LRIs, stratified by Socio-demographic Index (SDI), pollution source, and demographics. Temporal trends were assessed using Joinpoint regression, with projections to 2050.

**Results:**

In 2021, PMP was responsible for an estimated 651,238 (95% UI, 121,605-1076,503) deaths and 29,098,331(6,988,265-48,127,683) DALY from LRIs globally (ASMR: 8.68; ASDR: 420.09 per 100,000). Despite significant declines since 1990 (ASMR EAPC: -2.44%), profound disparities persisted. Low-SDI regions experienced 23-fold higher mortality (ASMR: 31.28 per 100,000) than high-SDI regions (ASMR: 1.34 per 100,000), with slower improvement rates (EAPC: -3.41% vs -6.76% in high-middle SDI). A critical epidemiological transition emerged: APMP became predominant in high-income regions (approaching 100% of PMP burden), while HAP dominated in low-SDI settings (ASMR: 26.12 per 100,000). Regional heterogeneity was marked, with Central Sub-Saharan Africa bearing the highest HAP burden and minimal APMP improvement (EAPC: -0.24%). Children under 5 years of age group still accounted for the most PMP-related deaths, with the number decreasing from 851,458 (95% UI: 211,026 to 1,363,810) in 1990 to 201,078 (95% UI:61,817 to 337,232). In contrast, the APMP-related burden increased among adults aged 20 years and older. Strong inverse correlations between SDI and disease burden (r=-0.85, p<0.001) confirmed socioeconomic determinants. Projections suggest decelerating improvements post-2030.

**Conclusion:**

The global PMP reduction masks widening inequities between regions at different development stages. The divergent pollution profiles-HAP persistence in low-SDI regions versus APMP dominance in high-SDI regions-necessitate differentiated intervention strategies addressing both household energy transitions and ambient air quality regulations to achieve health equity by 2050.

## 1 Introduction

Lower respiratory infections (LRIs) are a global health challenge. In 2021, there were 344 million new cases of non-COVID-19 LRIs and 2.18 million deaths (Yang et al., 2022; GBD 2021 Lower Respiratory Infections and Antimicrobial Resistance Collaborators, 2024). Mortality rates are highest in adults older than 70 years and in children younger than 5 years (Falagas et al., 2008), and both incidence and mortality are generally higher in males. Risk factors for LRIs mortality in all age groups include exposure to tobacco smoke, indoor and outdoor particulate matter, and extreme temperatures (GBD 2019 LRI Collaborators, 2022).

Particulate matter pollution (PMP) is a ubiquitous form of air pollution. PMP can be divided into household air pollution (HAP) and ambient particulate matter pollution (APMP) (Chen et al., 2024). PMP poses a significant health threat to individual suffering from lower respiratory infections, exacerbating their condition and contributing to the global burden of these diseases (Horne et al., 2018). Over the past few decades, there has been a global shift in the relative contributions of HAP and APMP (Kang et al., 2023). The varying trajectories of HAP and APMP have varied distinctively across regions (Thi Khanh et al., 2024). HAP is mainly caused by the burning of solid fuels (such as wood and coal) in households, which is still the main source of energy in many developing countries (Hammer et al., 2020). Despite the strict pollution control measures taken by some developed countries, APMP is still increasing in many developing countries (Shupler et al., 2018). According to the latest GBD 2021 risk factor study, HAP is identified as the risk factor contributing the most significantly to the disease burden of LRIs (Christian and Cornelia, 2024). The high use of fossil fuels in developing countries may be one of the reasons why HAP has a significant impact on LRIs (Ahmed et al., 2016).

Although PMP continues to pose a threat to global public health, the impact of PMP on LRI after the data update in 2021 has not been analyzed (Li et al., 2022). Moreover, previous studies have mainly examined the association between specific types of LRIs and PMP. They have neither considered the effects of specific PMP subtypes on various LRIs nor conducted an in - depth examination of how these subtypes affect individuals across distinct regions, SDI levels, age brackets, and genders (Hu et al., 2023; Wu et al., 2021). Hu et al. conducted a study on the effect of PM_2.5_ air pollution on the global burden of LRIs using the data from the GBD 2019. However, since the GBD 2019 data were collected before the COVID-19 pandemic, the impact of PM_2.5_ air pollution on the global burden of LRIs during this pandemic remains unanalyzed (Hu et al., 2023).

This study seeks to thoroughly investigate the effects of PMP, which includes both HAP and APMP on LRIs using the updated GBD 2021 data. It specifically examines the LRIs burden at a global, regional, and national scale from 1990 to 2021 and projects potential trends up to 2050 using various modeling techniques.

## 2 Methods

### 2.1 Overview

The GBD 2021 study, a sweeping and in-depth research endeavor, delivers a thorough examination of the worldwide, regional, and national impact of diseases, injuries, and risk factors. Directed by the Institute for Health Metrics and Evaluation (IHME), this study encompasses an evaluation of more than 300 conditions and injuries in 204 nations and territories, spanning the years 1990 to 2021 (GBD 2021 Diseases and Injuries Collaborators, 2024). By examining vital indicators such as incidence, prevalence, death rates, and disability-adjusted life years (DALYs), the GBD 2021 study sheds light on the shifting dynamics and patterns of health loss across the globe. The Socio-Demographic Index (SDI) is a composite measure that integrates data on income, education, and fertility rates to gauge the developmental status of a region or country.

This index is instrumental in categorizing regions according to their developmental stages. SDI divides regions into five distinct groups: low, low-middle, middle, high-middle, and high. These groups represent a developmental gradient from the least developed to the most developed areas (scaled from 0 to 100). The low SDI indicates populations that are poorer and less educated, while the high SDI represents wealthier and more educated societies (Li et al., 2018; GBD 2021 Risk Factors Collaborators, 2024; Li et al., 2024). In the 11th revision of the International Classification of Diseases (ICD-11), the code for lower respiratory infection is CA22.1.

### 2.2 Exposure assessment

The GBD database offers extensive evaluations of the risk factors that contribute to the global disease burden, with a focus on particulate matter pollution. Household air pollution from solid fuels (HAP) is characterized by exposure to high levels of particulate matter and other pollutants resulting mainly from the indoor combustion of solid fuels like wood, agricultural waste, and animal dung for cooking and heating purposes (Christian and Cornelia, 2024). The GBD 2021 HAP Collaborators estimated HAP exposure and trends and attributable burden for ten diseases including lower respiratory infections for 204 countries and territories from 1990 to 2021. They first estimated the mean fuel type-specific concentrations (in μg/m^3^) of fine particulate matter (PM_2·5_) pollution to which individuals using solid fuels for cooking were exposed, categorized by fuel type, location, year, age, and sex. Using a systematic review of the epidemiological literature and a newly developed meta-regression tool (meta-regression: Bayesian, regularized, trimmed), they derived disease-specific, non-parametric exposure-response curves to estimate relative risk as a function of PM_2·5_ concentration. They combined our exposure estimates and relative risks to estimate population attributable fractions and attributable burden for each cause by sex, age, location, and year (GBD 2019 Diabetes and Air Pollution Collaborators, 2022).

In contrast, APMP also referred to as ambient PM_2.5,_ is quantified by combining ground-level monitoring data with satellite-derived measurements (Christian and Cornelia, 2024). For estimation of ambient air pollution exposure, we used the Data Integration Model for Air Quality (DIMAQ2). This model integrates data from satellite-based measurements of aerosol optical depth, ground measurements from 9960 PM monitoring stations across 108 countries, and chemical transport model simulations (Christian and Cornelia, 2024). Global values of PM_2·5_, provided at a 0·1° × 0·1° resolution, were population-weighted to generate mean exposure for each location. Methods and data sources are described in more detail in the other article and have been published elsewhere (Shaddick et al., 2018; GBD 2019 Diabetes and Air Pollution Collaborators, 2022; Christian and Cornelia, 2024).

In this context, PMP is an overarching term that includes both HAP and APMP. It underscores the health risks associated with the inhalation of fine particles (PM_2.5_), which can deeply infiltrate the lungs and translocate into the bloodstream, causing a spectrum of cardiovascular and respiratory diseases (Christian and Cornelia, 2024).

### 2.3 Burden estimation

To facilitate comparisons of mortality and disease burden across various populations and timeframes, age-standardized death rates and Disability-Adjusted Life Year (DALY) rates were determined. Age-standardized death rates were calculated by applying the age-specific death rates of a particular year to a standardized population, thus allowing for comparisons that account for differences in population age structures. The DALY rates, which also account for age standardization, were used to assess the overall disease burden, considering both the years of life lost to early death and the years lived with disability. These standardized rates help in identifying trends and health outcome disparities over time.

### 2.4 Statistical analysis

The temporal trend was evaluated using the Joinpoint Regression Program (Version 5.0.2), and the Estimated Annual Percent Change (EAPC) was calculated during 1990–2021 with default parameters. The EAPC quantifies the trends in these age-standardized rates, calculated through a regression model on the log-transformed rates to determine the annual percentage change, reflecting the temporal changes in disease burden and mortality(Version 5.0.2) (Ahmed et al., 2016). Statistical analyses and the visualization of results were conducted using the R software (version 4.3.1, R Core Team).

### 2.4 Projection analysis

The Bayesian Age-Period-Cohort (BAPC) model was used to forecast future disease burden by considering the effects of age, period, and cohort, providing essential insights for public health strategies and resource allocation (GBD 2021 Risk Factors Collaborators, 2024). This study employs a hierarchical Age-Period-Cohort (APC) model to disentangle the underlying temporal patterns in mortality data into age, period, and cohort effects. The analysis utilizes two data sources: observed age-specific mortality data from 1980 to 2021 and forecasted data from 2022 to 2050, enabling the examination of both historical trends and future scenarios. To address the well-known identifiability problem inherent in APC models, we implemented a Bayesian framework with the following features: (1) parameters were constrained to ensure model stability and interpretability; (2) flexible smoothing was achieved using quintic B-splines for both the age and period dimensions; and (3) hierarchical intrinsic Gaussian Markov Random Field (iGMRF) priors were applied to preserve smoothness across adjacent age groups and time periods. Parameters were estimated using Markov Chain Monte Carlo (MCMC) simulation to approximate the posterior distributions. Finally, the model’s reliability was assessed through leave-one-out cross-validation to evaluate its robustness and predictive performance.

## 3 Results

### 3.1 Global reduction of PMP-related burden masks regional disparities

**Figure 1:**
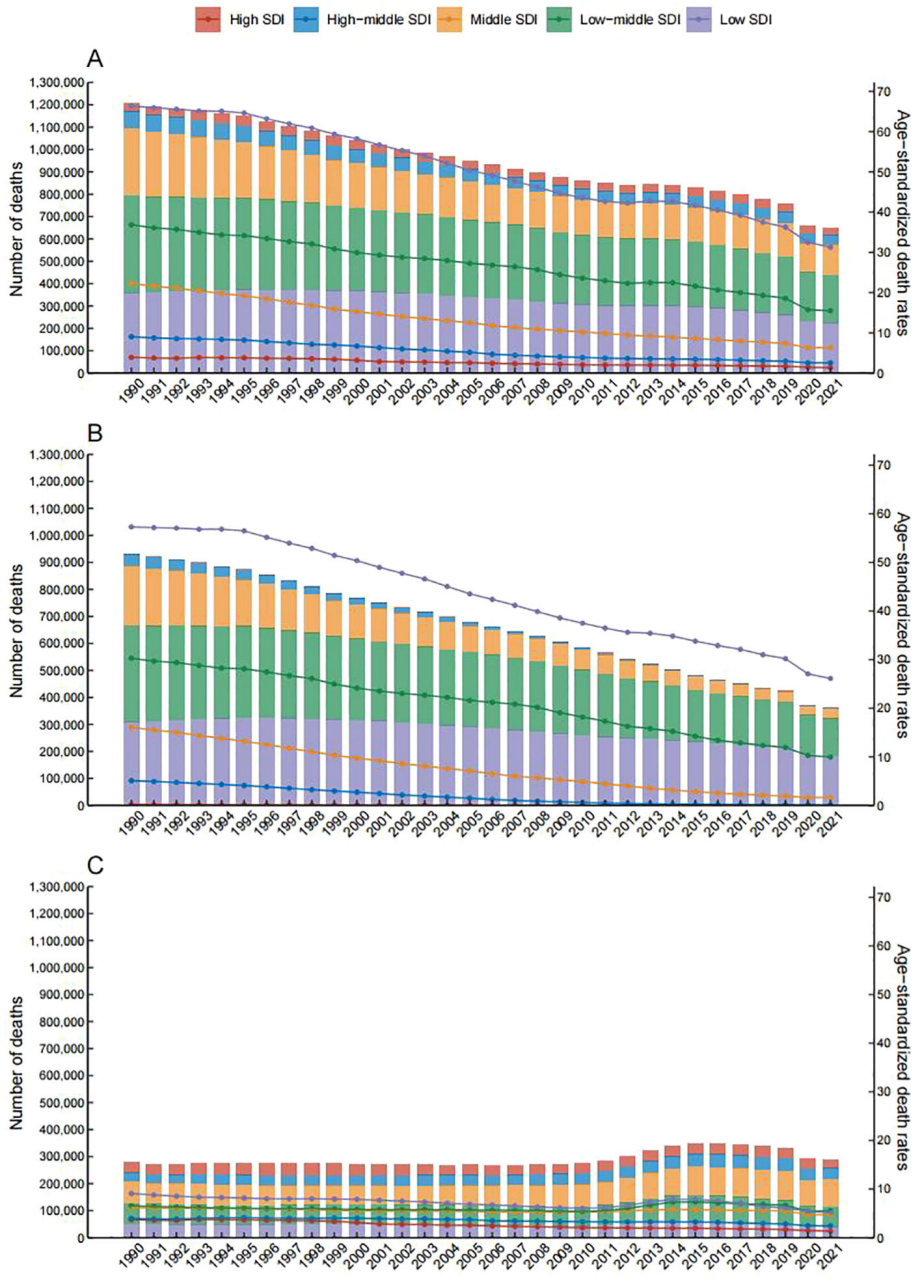
Global number of deaths and age-specific rates of lower respiratory infections attributable to particulate matter pollution, by five Socio-demographic Index (SDI) levels, 1990–2021. **(A)** Total particulate matter pollution. **(B)** Household air pollution. **(C)** Ambient particulate matter pollution. Bars represent death counts; lines represent age-standardized rates.

In 2021, PMP accounted for approximately 0.65 million deaths and 29.1 million DALYs linked to LRIs globally, with age-standardized mortality and DALY rates (ASMR: 8.68 [95% UI: 1.71–14.39]; ASDR: 420.09 [95% UI: 106.35–693.12] per 100,000 population) (**Supplementary Tables S1, S4**). Over the past three decades, PMP-attributable LRIs mortality declined significantly (**Figure 1A**). Despite a marked reduction in the proportional burden of HAP since 1990, HAP remained a larger contributor to the global LRIs burden in 2021 compared to APMP (**Figure 1B**). Annual declines in PMP-related DALYs were driven predominantly by reductions in HAP (**Supplementary Figures S1A, B**, **Supplementary Tables S4, S5**), whereas APMP-associated DALYs fluctuated, peaking between 2010 and 2015 before declining (**Supplementary Figure S1C**, **Supplementary Table S6**). In 2020 and 2021, compared with the period before 2019 (prior to the COVID-19 pandemic), the contributions of PMP, APMP, and HAP to LRIs decreased significantly (**Figure 1A**, **Supplementary Figure S1**).

By 2021, PMP contributed substantially to LRIs mortality and DALYs in middle-, low-middle-, and low-SDI regions, while having minimal impact in high- and high-middle-SDI regions (**Figure 1A–C**, **Supplementary Figure S1**). A clear gradient emerged across SDI regions for both mortality and DALY rates (per 100,000 population). High-SDI regions exhibited the lowest burden across all pollution types: PMP showed an ASMR of 1.34 (95% UI: 0.15 to 2.5) and ASDR of 25.79 (95% UI: 3.14 to 47.22) per 100,000 population, while HAP had an ASMR of 0(95% UI: 0 to 0.04) and ASDR of 0.1(95% UI: 0 to 0.89) per 100,000 population. APMP burden was similar to total PMP, with an ASMR of 1.33 (95% UI: 0.15 to 2.5) and ASDR of 25.68 (95% UI: 3.16 to 47.16) per 100,000 population. In contrast, low-SDI regions recorded the highest disease burden. PMP-related rates reached an ASMR of 31.28 (95% UI: 5.61 to 49.7) and ASDR of 1,141.25 (95% UI: 252.52 to 1,851.51) per 100,000 population. HAP showed similarly high rates with an ASMR of 26.12 (95% UI: 4.9 to 42.78) and ASDR of 942.41 (95% UI: 224.28 to 1,579.8) per 100,000 population. The lowest APMP-associated ASDR was observed in low-middle-SDI regions at 203.51 (95% UI: 48.16 to 381.55) per 100,000 population (**Supplementary Tables S1, S2**).

Temporal trends further highlighted inequities. High-middle-SDI regions achieved the steepest ASMR declines for PMP (EAPC: −6.76 [95% CI: −6.99 to −6.54]), followed by middle-SDI regions (−5.44 [−5.56 to −5.33]). High-SDI regions showed pronounced reductions in HAP (EAPC: −13.81 [−14.15 to −13.48]) and APMP (EAPC: −3.44 [−3.61 to −3.28]) (**Supplementary Tables S2, S3**). In contrast, low-SDI regions lagged, with smaller annual improvements: PMP (EAPC: −3.41 [−3.58 to −3.23]), HAP (−3.65 [−3.83 to −3.48]), and APMP (−2.12 [−2.46 to −1.77]). Similar patterns were observed for ASDR across SDI regions.

### 3.2 Regional variations in etiological shifts of lower respiratory infection mortality and DALYs

**Figure 2:**
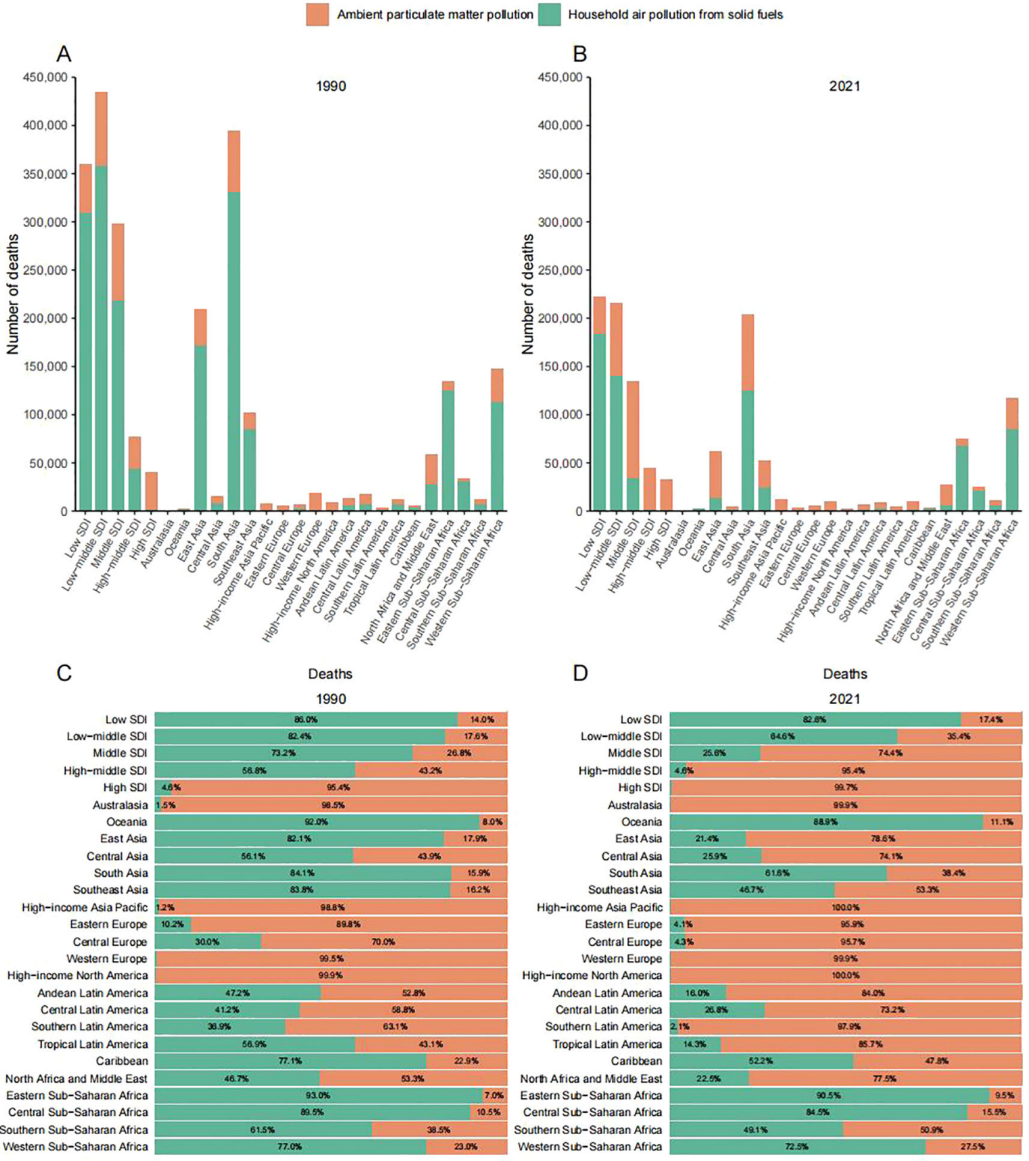
Deaths from lower respiratory infections attributable to household air pollution (HAP) and ambient particulate matter pollution (APMP), by 21 GBD regions and five SDI levels, in 1990 **(A)** and 2021 **(B)**. Proportional distributions are shown for 1990 **(C)** and 2021 **(D)**. Orange represents APMP; green represents HAP.

Globally, APMP-attributable proportional mortality and DALYs demonstrated a sustained increase across all GBD regions between 1990 and 2021, with the most pronounced escalation observed in East Asia (proportional mortality: 17.9% [1990] to 78.6% [2021]; DALYs: 17.1% [1990] to 76.4% [2021]) (**Figures 2C, D**, **Supplementary Figures S2C, D**). By 2021, APMP emerged as the predominant etiology of LRIs-associated mortality in high-income regions, accounting for 100% of deaths in High-Income Asia Pacific and High-Income North America, and 99.9% in Western Europe and Australasia (**Figures 2A, B**). Concurrently, ASMR and ASDR for HAP approached near-elimination thresholds in Eastern Europe, High-Income Asia Pacific, Australasia, and High-Income North America. Notably, High-Income North America exhibited the steepest decline in HAP burden, with an EAPC of −23.38 (95% CI: −24.67 to −22.06) (**Figures 2E**, **Supplementary Tables S1–S6**).

In contrast, Central Sub-Saharan Africa bore the highest ASMR and ASDR for PMP and HAP, while Southern and Western Sub-Saharan Africa recorded peak ASMR and ASDR for APMP, respectively. Temporal trends revealed heterogeneous progress: Southern Sub-Saharan Africa demonstrated the slowest ASMR decline for PMP (EAPC =-1.82, 95% CI: -1.97 to -1.66), while Central Sub-Saharan Africa showed minimal improvement for APMP (EAPC = −0.24, 95% CI: −0.46 to −0.02). Similarly, Oceania exhibited the lowest ASMR reduction for HAP (EAPC =-1.76, 95% CI: -1.84 to -1.69). Southern Sub-Saharan Africa further displayed the slowest ASDR declines across all etiologies: PMP (EAPC = -1.5, 95% CI: -1.61 to -1.4), HAP (EAPC =-2.04, 95% CI: -2.15 to -1.92) and APMP (EAPC = -0.42, 95% CI: -0.52 to -0.31).

### 3.3 Global map of EAPC in age-standardized death and DALY rates due to particulate pollution

**Figure 3:**
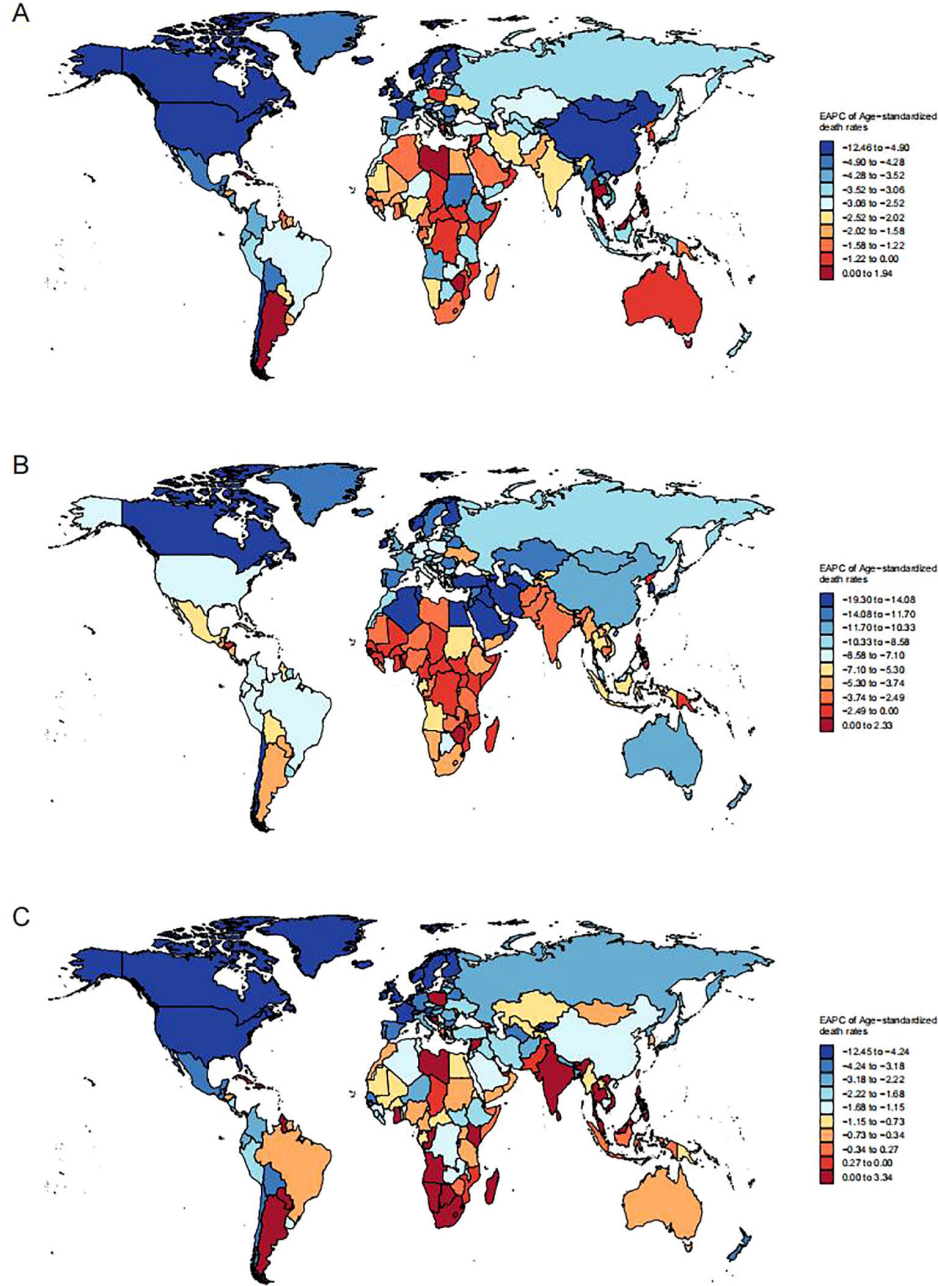
Global distribution of the Estimated Annual Percent Change (EAPC) in age-standardized death rates from lower respiratory infections attributable to **(A)** total particulate matter pollution (PMP), **(B)** household air pollution (HAP), and **(C)** ambient particulate matter pollution (APMP), 1990–2021.

The global trend for mortality from LRIs due to PMP is -2.44 (95% CI, -2.51 to -2.37), with HAP and APMP contributing trends of -2.56 (95% CI, -2.65 to -2.47) and -1.98 (95% CI, -2.29 to -1.67) respectively (**Figure 3A** and **Table 1**). In most countries worldwide, both the number of deaths and mortality rates due to PMP are declining. However, in Argentina, there has been a concerning escalation in mortality from LRIs attributed to PMP. The death toll has risen from 1452 (95% UI: 207-2870) in 1990 to 3879 (95% UI: 445-7704) in 2021. with the ASMR also rising from 4.99 (95% UI: 0.7-9.86) to 6.72 (95% UI: 0.77-13.31) (**Supplementary Table S7**).

**Table 1:** EAPC of age-standardized death rates of lower respiratory infection due to particulate matter pollution in 5 SDI and 21 GBD regions.

Characteristics	EAPC of age-standardized death rates (95% CI) due to		
	Particulate matter pollution	Household air pollution from solid fuels	Ambient particulate matter pollution
Global	-2.44 (-2.51–2.37)	-2.56 (-2.65–2.47)	-1.98 (-2.29–1.67)
Female	-2.28 (-2.35–2.22)	-2.41 (-2.51–2.31)	-1.74 (-2.04–1.44)
Male	-2.63 (-2.73–2.52)	-2.73 (-2.83–2.63)	-2.23 (-2.56–1.9)
Low SDI	-1.99 (-2.08–1.9)	-2.16 (-2.26–2.07)	-0.68 (-0.95–0.42)
Low-middle SDI	-2.48 (-2.65–2.31)	-3.21 (-3.46–2.97)	-0.13 (-0.47-0.22)
Middle SDI	-8.45 (-8.71–8.2)	-12.12 (-12.5–11.74)	-4.79 (-5.05–4.53)
High-middle SDI	-10.03 (-10.64–9.42)	-20.07 (-21.11–19.02)	-6.52 (-6.95–6.08)
High SDI	-8.46 (-8.91–8.01)	-23.76 (-24.58–22.93)	-7.58 (-7.9–7.26)
East Asia	-12.91 (-13.53–12.28)	-15.79 (-16.6–14.96)	-7.9 (-8.34–7.46)
Southeast Asia	-4.82 (-4.88–4.76)	-5.63 (-5.78–5.48)	-1.88 (-2.13–1.63)
Oceania	-1.67 (-1.75–1.59)	-1.76 (-1.84–1.69)	-0.66 (-1–0.32)
Central Asia	-2.62 (-3.07–2.17)	-4.37 (-5.21–3.54)	-0.21 (-0.59-0.18)
Central Europe	-9.51 (-9.87–9.15)	-14.41 (-15.13–13.69)	-7.47 (-7.79–7.15)
Eastern Europe	-10.27 (-10.85–9.69)	-15.31 (-16.53–14.06)	-9.78 (-10.36–9.19)
High-income Asia Pacific	-11.49 (-12.22–10.75)	-23.38 (-24.67–22.06)	-11.43 (-12.16–10.69)
Australasia	-3.4 (-4.65–2.14)	-12.77 (-14.4–11.11)	-3.35 (-4.59–2.09)
Western Europe	-7.22 (-7.37–7.08)	-14.38 (-15.15–13.6)	-7.2 (-7.34–7.06)
Southern Latin America	-6.79 (-7.67–5.9)	-14.8 (-15.57–14.03)	-5.27 (-6.04–4.49)
High-income North America	-5.26 (-5.65–4.87)	-7.87 (-8.44–7.29)	-5.26 (-5.65–4.88)
Caribbean	-0.98 (-1.18–0.79)	-0.94 (-1.12–0.75)	-1.33 (-1.68–0.99)
Andean Latin America	-8.41 (-8.74–8.09)	-9.99 (-10.26–9.72)	-7.45 (-7.93–6.97)
Central Latin America	-8.93 (-9.33–8.54)	-8.24 (-8.41–8.07)	-9.5 (-10.11–8.89)
Tropical Latin America	-6.46 (-6.7–6.22)	-9.63 (-9.81–9.45)	-3.92 (-4.16–3.67)
North Africa and Middle East	-2.11 (-2.54–1.67)	-4.76 (-5.57–3.93)	-1.74 (-2.18–1.3)
South Asia	-3.09 (-3.24–2.94)	-3.77 (-4.02–3.52)	-0.35 (-0.67–0.02)
Central Sub-Saharan Africa	-4 (-4.32–3.69)	-4.14 (-4.48–3.8)	-2.66 (-2.82–2.5)
Eastern Sub-Saharan Africa	-2.09 (-2.16–2.03)	-2.19 (-2.25–2.12)	-0.72 (-0.94–0.51)
Southern Sub-Saharan Africa	-1.82 (-1.97–1.66)	-2.01 (-2.18–1.84)	-1.34 (-1.52–1.16)
Western Sub-Saharan Africa	-2.12 (-2.22–2.02)	-2.4 (-2.53–2.27)	-1.04 (-1.38–0.7)

The general trend in most countries indicates a decline in both the number of deaths and mortality rates due to HAP and APMP. However, an upward trend in deaths from LRIs due to HAP is observed specifically in Zimbabwe and Lesotho, with increases of 1.4 (95% CI: 0.76-2.05) and 0.77 (95% CI: 0.16-1.38) respectively (**Figure 3B** and **Supplementary Table S8**). As for APMP, an increasing trend is noted in 49 countries and regions, with Cabo Verde recording the most substantial rise at 3.34 (95% CI: 2.68-4.01) (**Figure 3C** and **Supplementary Table S9**).

Globally, DALYs for patients with LRIs attributed to PMP have exhibited a decreasing trend, with a rate of -2.32 (95% CI: -2.39 to -2.25). Specifically, DALY due to HAP showed a trend of -2.5 (95% CI: -2.6 to -2.41), while those due to APMP were -1.63 (95% CI: -1.93 to -1.34) (**Table 2**). In contrast to the global trend, DALYs attributed to PMP in Argentina, Lesotho, Kuwait, and Zimbabwe have shown an increasing trend (**Supplementary Figure S3A**, **Supplementary Table S10**). Similarly, DALYs due to HAP have risen in Lesotho, the Northern Mariana Islands, and Zimbabwe (**Supplementary Figure S3B**, **Supplementary Table S11**). APMP has contributed to an upward trend in DALYs in 22 countries, with Lesotho witnessing the most significant increase at 1.89 (95% CI: 1.49-2.29) (**Supplementary Figure S3C**, **Supplementary Table S12**).

**Table 2:** EAPC of age-standardized DALYs rates of lower respiratory infection due to particulate matter pollution in 5 SDI and 21 GBD regions.

Characteristics	EAPC of age-standardized DALY rates (95% CI) due to		
	Particulate matter pollution	Household air pollution from solid fuels	Ambient particulate matter pollution
Global	-2.32 (-2.39–2.25)	-2.5 (-2.6–2.41)	-1.63 (-1.93–1.34)
Female	-2.18 (-2.24–2.12)	-2.36 (-2.47–2.26)	-1.45 (-1.73–1.17)
Male	-2.48 (-2.58–2.38)	-2.67 (-2.77–2.56)	-1.82 (-2.13–1.51)
Low SDI	-1.93 (-2.01–1.84)	-2.11 (-2.2–2.01)	-0.62 (-0.88–0.35)
Low-middle SDI	-2.28 (-2.43–2.14)	-3.05 (-3.28–2.82)	0.15 (-0.17-0.48)
Middle SDI	-7.22 (-7.48–6.96)	-10.65 (-10.94–10.36)	-3.69 (-3.96–3.4)
High-middle SDI	-8.45 (-9.02–7.88)	-17.94 (-18.79–17.09)	-5.02 (-5.42–4.62)
High SDI	-3.03 (-3.19–2.87)	-17.97 (-18.59–17.35)	-2.63 (-2.73–2.54)
East Asia	-10.99 (-11.51–10.46)	-14.42 (-15.12–13.72)	-5.59 (-5.95–5.22)
Southeast Asia	-2.29 (-2.37–2.21)	-3.4 (-3.62–3.17)	0.29 (0.1-0.48)
Oceania	-0.11 (-0.15–0.07)	-0.19 (-0.22–0.15)	0.71 (0.48-0.95)
Central Asia	-2.6 (-3.04–2.15)	-4.37 (-5.19–3.53)	-0.18 (-0.56-0.21)
Central Europe	-5.56 (-5.74–5.37)	-12.13 (-12.77–11.48)	-3.5 (-3.72–3.27)
Eastern Europe	-9.51 (-10.07–8.93)	-14.52 (-15.71–13.32)	-9.01 (-9.59–8.43)
High-income Asia Pacific	-1.28 (-1.64–0.91)	-12.97 (-14.25–11.66)	-1.22 (-1.58–0.86)
Australasia	-0.26 (-0.59-0.08)	-10.33 (-11.01–9.65)	-0.2 (-0.53-0.13)
Western Europe	-2.95 (-3.07–2.84)	-10.66 (-11.31–10)	-2.94 (-3.05–2.82)
Southern Latin America	-1.82 (-2.11–1.54)	-10.18 (-10.57–9.79)	-0.68 (-0.95–0.42)
High-income North America	-2.71 (-2.85–2.58)	-5.77 (-6.02–5.52)	-2.71 (-2.85–2.57)
Caribbean	-0.55 (-0.72–0.38)	-0.68 (-0.86–0.5)	-0.01 (-0.2-0.19)
Andean Latin America	-6.77 (-7.05–6.49)	-8.89 (-9.16–8.62)	-5.59 (-6.02–5.16)
Central Latin America	-8.14 (-8.44–7.84)	-7.76 (-7.91–7.6)	-8.43 (-8.87–7.99)
Tropical Latin America	-4.45 (-4.57–4.33)	-7.55 (-7.74–7.36)	-2.03 (-2.15–1.91)
North Africa and Middle East	-1.62 (-1.96–1.28)	-3.81 (-4.3–3.32)	-1.2 (-1.57–0.83)
South Asia	-2.72 (-2.83–2.6)	-3.45 (-3.67–3.23)	0.12 (-0.23-0.48)
Central Sub-Saharan Africa	-3.84 (-4.14–3.54)	-3.99 (-4.32–3.66)	-2.43 (-2.59–2.27)
Eastern Sub-Saharan Africa	-2.03 (-2.09–1.97)	-2.12 (-2.18–2.06)	-0.65 (-0.86–0.43)
Southern Sub-Saharan Africa	-1.5 (-1.61–1.4)	-2.04 (-2.15–1.92)	-0.42 (-0.52–0.31)
Western Sub-Saharan Africa	-2.06 (-2.16–1.97)	-2.35 (-2.48–2.22)	-0.96 (-1.3–0.62)

### 3.4 Correlation of particulate pollution-related disease burden with SDI in 21 GBD regions and 204 countries and territories

**Figure 4:**
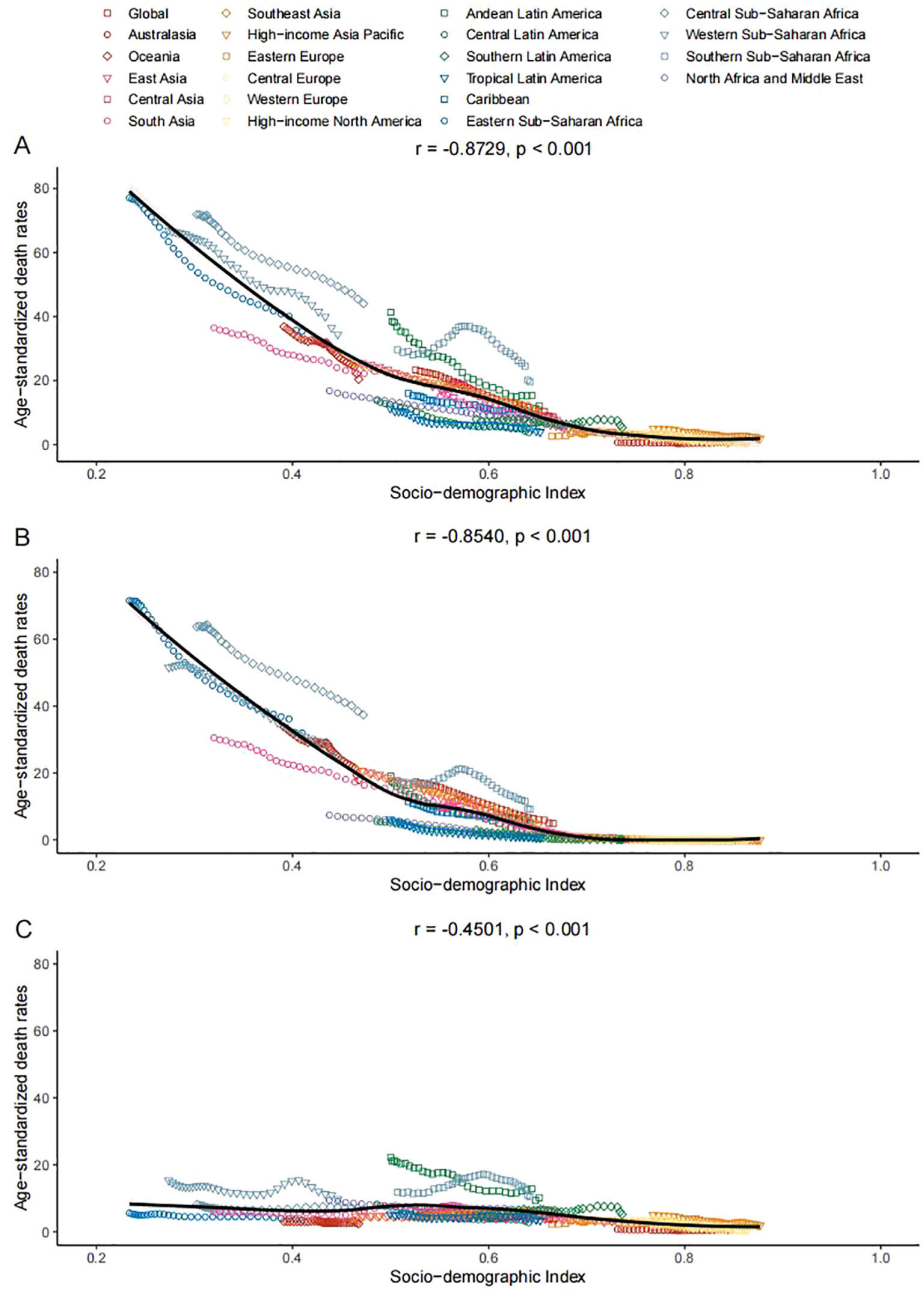
Association between age-standardized death rates and socio-demographic index for lower respiratory infections due to **(A)** PMP, **(B)** HAP, and **(C)** APMP, across 21 GBD regions, 1990–2021.

Between 1990 and 2021, within the 21 GBD regions, there was a consistent decrease in age-standardized death rates of LRIs attributable to PMP and SDI levels rose. This relationship was highly significant, with a Spearman’s rank correlation coefficient (r) of -0.8729, denoting a strong, statistically significant inverse correlation (p<0.001). HAP, a major part of PMP, also showed a downward trend with an r value of -0.8540 (p<0.001). APMP, another aspect of PMP, likewise exhibited a reduction in age-standardized mortality rates with increasing SDI, but the correlation was less strong, with an r value of -0.4501 (p<0.001) (**Figure 4**).

DALYs for LRIs patients have shown a trend that reflects the patterns seen in death rates, with a decline as SDI increases. The correlation for PMP was highly significant, with an r value of -0.8877 (p<0.001). HAP exhibited a marked decrease with an increase in SDI, with an r value of -0.8690 (p<0.001). APMP also displayed a downward trend with increasing SDI, though less markedly, with an r value of -0.5577 (p<0.001) (**Supplementary Figure S4**).

**Figure 5:**
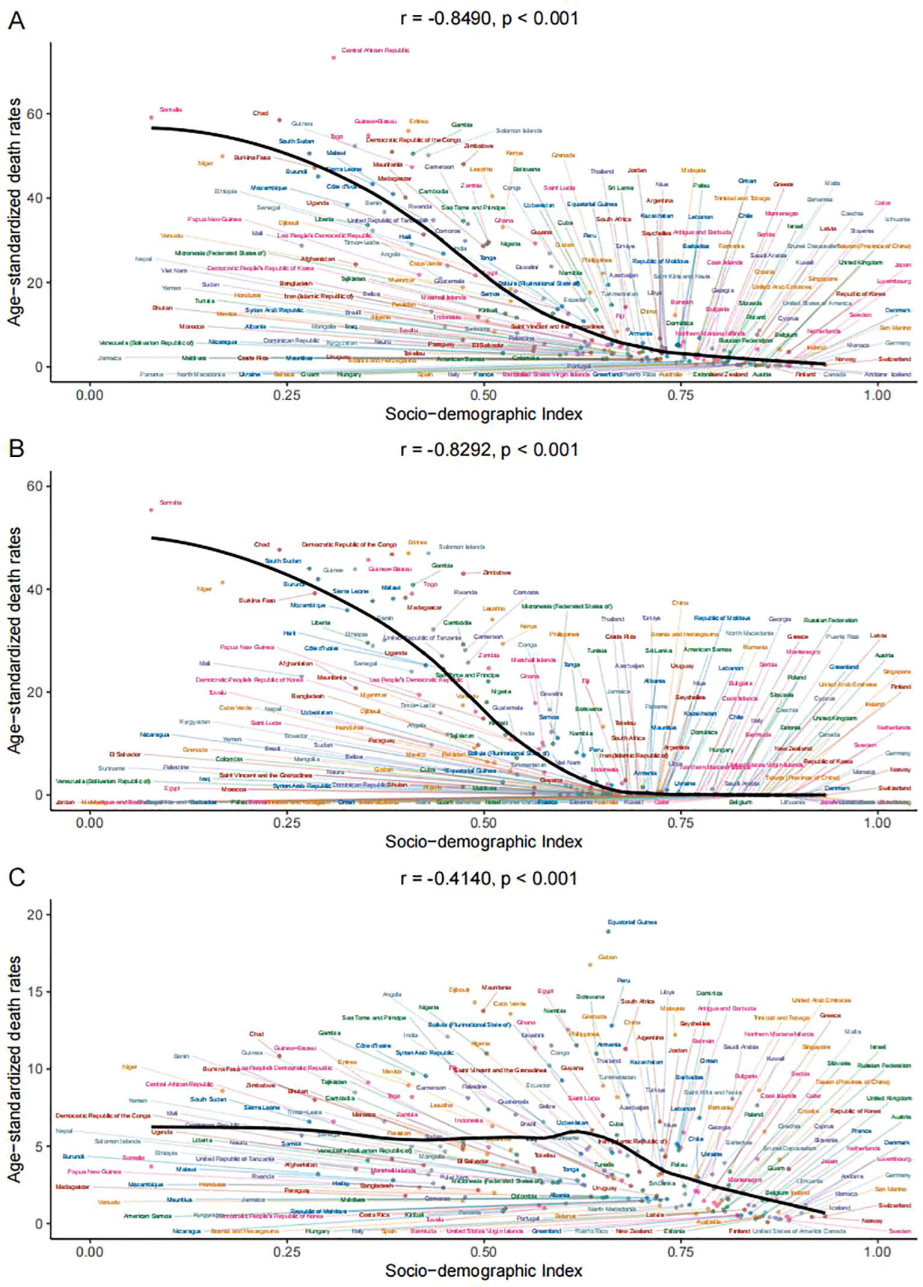
Association between age-standardized death rates and socio-demographic index for lower respiratory infections due to **(A)** PMP, **(B)** HAP, and **(C)** APMP, across 204 countries and territories, 1990–2021.

In 2021, global age-standardized death rates for LRIs caused by PMP decline with increasing SDI, with a correlation coefficient (r) of -0.8490 (p<0.001). Both HAP and APMP contributed to this trend, with r values of -0.8292 and -0.4140, respectively (**Figure 5**). DALY rates mirrored death rates, showing strong negative correlations with SDI for PMP (r =-0.8576, p < 0.001), HAP (r = -0.8327, p<0.001), and APMP (r= -0.5252, p<0.001) (**Supplementary Figure S5**).

### 3.5 Gender and age-related disparity in global burden from particulate pollution

**Figure 6:**
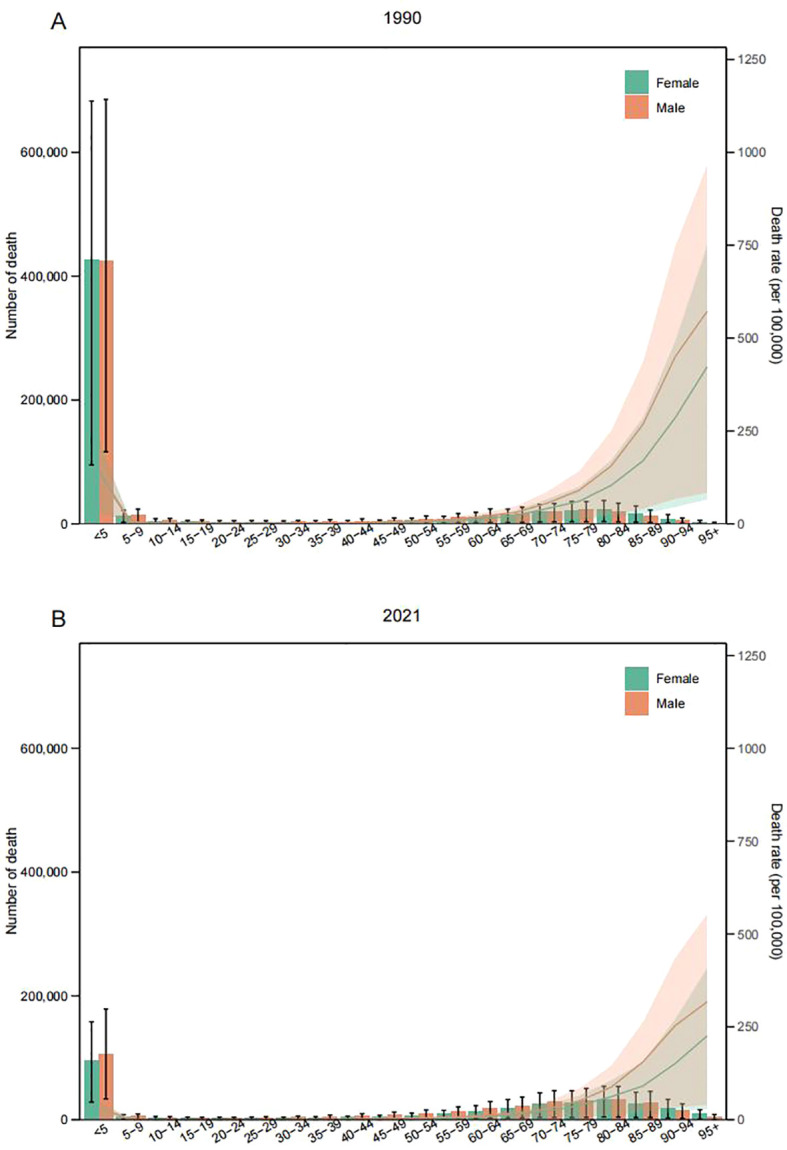
Number of deaths (bar chart) and death rates (line chart) of lower respiratory infections attributable to particulate matter pollution, by age group and sex, in 1990 **(A)** and 2021 **(B)**.

Most deaths from LRIs due to PMP occur in children under 5 years old. In 2021, there was a significant drop in deaths from PMP compared to 1990. The 0–4 age group still accounted for the most PMP-related deaths, with the number decreasing from 851,458.04 (95% UI: 211,026 to 1,363,810) in 1990 to 201,078 (95% UI, 618,178 to 337,232) (**Figure 6**). In 2021, there was a significant drop in deaths from HAP compared to 1990, with the 0–4 age group still accounting for the most HAP-related deaths (from 686,641 to 142,917) (**Supplementary Figure S6**). Deaths attributed to APMP decreased in the 0–4 age group but increased for those aged 20 and above in 2021 compared to 1990, with males having more APMP-attributed deaths than females before the age of 90 (**Supplementary Figure S7**).

DALYs for PMP-caused LRIs significantly decreased in the 0–4 age group from 1990 to 2021, with similar decreases for HAP and APMP within this age group. However, APMP-caused DALYs increased in those aged 20 and above in 2021 compared to 1990 (**Supplementary Figures S8**-**S10**).

### 3.6 Projected disease burden for lower respiratory infections from 2022 to 2050

**Figure 7:**
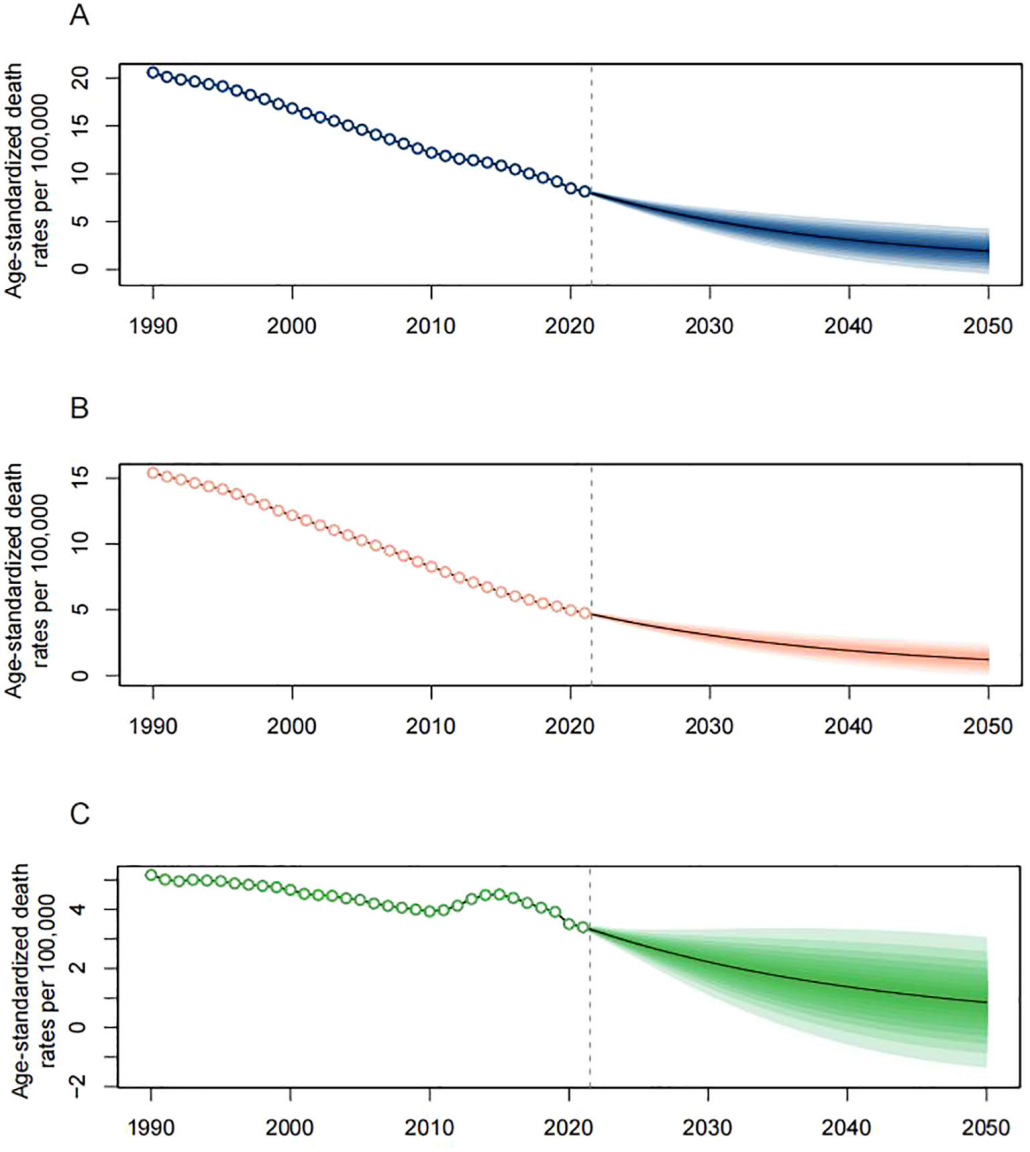
Projected global age-standardized death rates from lower respiratory infections attributable to **(A)** PMP, **(B)** HAP, and **(C)** APMP, 2022–2050.

The projected mortality rate for LRIs due to PMP is expected to drop sharply from 2022 to 2030, after which the decline is projected to slow (**Figure 7A**). HAP, which has seen a rapid decrease in mortality rates from 1990 to 2021, is predicted to maintain this downward trend through 2050, albeit at a slower rate (**Figure 7B**). For APMP, the morality rate is also expected to decrease after 2022, but with less certainty due to wider confidence intervals in the estimates (**Figure 7C**).

## 4 Discussion

By analyzing trends from 1990 to 2021, this study provides a comprehensive assessment of the burden of LRIs attributable to PMP, capturing both long-term dynamics and the recent disruptions caused by the COVID-19 pandemic. This extended temporal scope allows for the identification of key transitional patterns, such as the point at which APMP surpassed HAP as the leading contributor to the PMP-related LRI burden in many regions, while also contextualizing the unique fluctuations observed during the 2020–2021 period within a broader historical trend (Hu et al., 2023).

During the COVID-19 pandemic, there were notable changes related to air pollution and the diagnosis and reporting of LRIs. In 2020 and 2021, compared with the period before 2019 (prior to the COVID-19 pandemic), the contributions of PMP, APMP, and HAP to LRIs decreased significantly (**Figure 1A**, **Supplementary Figure S1**). A study by Meina Zheng et al., which analyzed the daily air pollution data of 596 major cities in 77 countries in 2020, found that the lockdown measures led to a 21% - 35% decrease in global NO_2_ concentrations and a 9% - 18% decrease in PM_2.5_ concentrations. The reduction in traffic flow, including the use of public transportation and private cars, was identified as an important factor contributing to this decline in air pollution (Zheng et al., 2025). Regarding LRIs, there were interferences in their diagnosis and reporting. The surge in COVID-19 cases caused a strain on medical resources, resulting in a decrease in the screening and reporting rates of LRIs like pneumonia, which may have led to an underestimation of the actual burden. Additionally, non-pharmaceutical interventions such as wearing masks and social distancing reduced the transmission of respiratory pathogens, potentially directly lowering the incidence of LRIs (Käding et al., 2024). However, since our data pertains to the results of air pollution research over the entire past 30 years, we were unable to conduct a more detailed analysis of the impact of COVID-19. This is also one of the limitations of our study.

While global age-standardized mortality and DALY rates for PMP-related LRIs have declined significantly over the past three decades, these trends mask stark regional disparities driven by socioeconomic development, environmental policies, and access to healthcare. A global decline in LRIs mortality, largely driven by significant reductions in deaths attributable to HAP (Burnett et al., 2018). The decrease can be credited to enhancements in air quality, diminished household pollution, advancements in public health, and socio-economic development (Solarin, 2020).

In regions with high SDI, the lower respiratory infection ASDR due to PMP significantly decreased from 90.29 (95% UI, 15.31-160.78) in 1990 to 25.79 (95% UI, 3.14-47.22) in 2021. In high-middle SDI regions, the drop was from 487.61 (95% UI, 107.88-812.87) to 64.5 (95% UI, 10.02-111.28). The reduction in HAP from solid fuels was particularly notable, with its contribution nearly vanishing in high SDI areas. It appears that the reduction in LRIs deaths related to HAP may be significantly influenced by the increased use of clean energy sources and efficient cooking technologies, which are likely contributing to the reduction of indoor air pollution (GBD 2021 Causes of Death Collaborators, 2024). Similarly, declining APMP burdens in these regions align with stringent air quality regulations and technological advancements in emission control (United Nations Economic Commission for Europe, 1999; Environmental Protection Agency, 2021). However, the disproportionate burden in low- and middle-SDI regions, particularly Sub-Saharan Africa and parts of Asia, highlights systemic inequities. The slower declines in PMP-related mortality and DALYs in these regions—exemplified by Central Sub-Saharan Africa’s stagnant APMP trends and Southern Sub-Saharan Africa’s minimal progress—emphasize gaps in infrastructure, healthcare access, and policy implementation. The persistence of HAP as a dominant contributor to LRIs in low-SDI settings points to ongoing reliance on solid fuels and biomass for cooking, perpetuating cycles of poverty and disease.

Contrary to the 2019 GBD estimates documenting 39 countries with rising APMP attributable DALYs, subsequent data through 2021 revealed a marked reduction to 22 nations exhibiting this trend (Hu et al., 2023). Argentina, a high-middle SDI country, has seen an increase in ASDR for LRIs due to PMP from 4.99 (95% UI: 0.7-9.86) in 1990 to 6.72 (95% UI: 0.77-13.31) in 2021. Conversely, ASDR due to HAP from solid fuels fell from 0.7 (95% UI: 0.03-2.88) to 0.12 (95% UI: 0-1), and for APMP, it rose from 4.29 (95% UI: 0.59-8.78) to 6.59 (95% UI: 0.76-13.29) (**Supplementary Tables S7–S9**). Argentina’s reduction in LRIs mortality from HAP aligns with documented clean energy policies including the PROBIOMASA biomass program (2013), RenovAr renewable energy auctions, and residential energy efficiency initiatives (Law Gratis, 2025; Argentina, 2025a; Argentina, 2025b; Chowdhury et al., 2023; Earthworks, 2024). The rise in APMP-related mortality coincides with expanded industrial operations in oil and gas sectors, documented high emissions at multiple processing facilities located near residential areas, and acknowledged enforcement gaps in existing environmental regulations (Castronuovo et al., 2025).

This pattern aligns with broader trends observed in countries experiencing rising SDI levels, where the reduction in LRIs mortality and DALYs attributed to HAP from solid fuels is more significant compared to APMP. The less pronounced decrease in APMP-related figures implies that pollution emissions from industrial and urban development may be more challenging to curtail rapidly, indicating a need for more intricate policies and technical strategies (Al-Janabi et al., 2021). The divergent trends between HAP and APMP impacts on LRIs mortality suggest that pollution sources tied to household activities are more amenable to rapid intervention, whereas curbing emissions from industrial and urban sectors requires multidimensional approaches integrating regulatory enforcement, technological innovation, and public awareness campaigns.

The differential health impacts of HAP and APMP underscore distinct challenges in mitigating LRIs. Targeted global interventions, such as promoting cleaner cooking technologies, have successfully reduced HAP-related mortality by addressing localized indoor pollution sources, as evidenced by declining burdens in multiple populations (Pratiti et al., 2020). In contrast, APMP emissions—driven by industrialization, urbanization, and transportation—reflect systemic challenges embedded in broader socio-economic development. These emissions demand multifaceted strategies integrating stringent regulations, technological innovations, and public engagement to curb long-term exposure risks (Burnett et al., 2018). While progress is achievable—as shown by effective urban pollution monitoring systems—APMP control remains slower than HAP reduction, partly due to its dependence on structural economic transitions. Most studies, including ours, treat HAP and APMP as independent risk factors. Emerging evidence, however, indicates that simultaneous exposure can produce synergistic effects on respiratory outcomes. A systematic review concluded that co-exposure to extreme PM_2.5_ and high temperature increases respiratory mortality by≈8%-substantially above the sum of individual effects (Anenberg et al., 2020). Integrated-exposure-response curves for infant LRIs are steepest at the very high PM_2.5_ concentrations characteristic of solid-fuel households, implying supra-additivity when ambient pollution is also elevated (Gordon et al., 2014). Mechanistically, endotoxin-rich HAP can prime airway inflammation, amplifying the epithelial damage and impaired bacterial clearance induced by ambient PM (Mendy et al., 2019). If such synergy operates, interventions that reduce only one source may yield smaller health gains than anticipated. Future cookstove trials should therefore measure personal HAP and ambient PM concurrently and test for interaction on LRIs risk, while policy makers should consider bundled clean-air strategies that address household and ambient pollution simultaneously.

Demographic vulnerabilities further complicate this disparity. For instance, older populations exhibit heightened sensitivity to both pollution types, yet APMP’s pervasive nature exacerbates mortality inequities in aging societies (Chen S. et al., 2024). Thus, prioritizing APMP mitigation requires policies tailored to high-risk groups and pollution hotspots, alongside scalable clean energy infrastructures to align environmental and public health goals.

Projections from the model indicate a sustained decrease in global lower respiratory infection death rates due to PMP, HAP, and APMP by 2050. This anticipated decline could be attributed to continuous national initiatives toward cleaner energy, stringent environmental policies, and technological innovations. Additionally, the proactive steps taken by the global community to address the health hazards of air pollution are likely to contribute to this positive trend (Zhang et al., 2022).

Our findings reveal a critical divergence in PMP-related LRIs burdens between low- and high-SDI regions, necessitating distinctly targeted interventions. In low-SDI regions, where HAP dominates, efforts should focus on accelerating accessible clean-energy transitions. This includes subsidizing clean cookstoves with user-centered financing models and integrating HAP reduction into broader rural development programs-such as pairing electrification projects with health-worker-led community awareness initiatives. In high- and middle-SDI regions, where APMP is the primary driver, policies must shift from mass-based PM_2.5_ control to toxicity-oriented mitigation. This involves: Enforcing Best Available Techniques in high-toxicity industries (e.g., metallurgy, shipping fuel standards); Establishing low-emission zones coupled with congestion pricing to curb traffic-sourced PM; Integrating carbon cost accounting and health impact assessments into urban and energy planning frameworks. Globally, we urge policymakers to adopt health-impact-weighted PM_2.5_ standards that account for toxic variability by source, and to strengthen cross-sector governance mechanisms-such as interdepartmental committees co-led by health and environment authorities—to ensure health outcomes are embedded in climate and energy decisions.

These projections are substantiated by the empirical foundation laid in this study, which fills a critical gap by assessing the overall burden of LRIs attributable to PMP, including HAP from solid fuels and APMP. By employing diverse statistical models and accounting for demographic and socio-economic factors (e.g., age, gender, geography, and Socio-demographic Index, SDI), this research not only quantifies current burdens but also provides a framework to validate future policy-driven health improvements. This study underscores the persistent global burden of PMP-attributable LRIs, marked by stark disparities across SDI strata and regions. Despite substantial declines in mortality and DALYs since 1990, low-SDI regions remain disproportionately affected, with HAP driving most of the burden in these settings. In contrast, APMP has emerged as the dominant contributor in high-income regions, reflecting shifts in pollution sources linked to industrialization and urbanization. The inverse correlation between SDI and PMP-related burden highlights the critical role of socioeconomic development in mitigating environmental health risks. However, rising APMP-attributable mortality in older age groups and certain countries (e.g., Argentina) signals emerging challenges, particularly in aging populations and regions with inadequate pollution controls. Projections suggest continued declines in PMP-related mortality, but progress may slow without targeted interventions, especially in low-resource settings. Prioritizing clean energy transitions (to reduce HAP) and stricter air quality regulations (to curb APMP) are essential to address inequities and sustain progress. Future efforts must also address sex- and age-specific vulnerabilities and monitor evolving trends in pollution-driven respiratory disease burdens globally.

However, the study has some limitations: (1) The GBD 2021 does not offer granular size-specific classifications of PMP, which restricts the analysis of epidemiological trends associated with different PMP particle sizes. (2) Assessments of PM_2.5_ exposure, which rely on modeled estimates instead of direct measurements at the individual level, might not comprehensively account for the localized or micro - environmental differences in pollution exposure. The pollution is not solely composed of PM_2.5_; it also encompasses other pollutants such as PM_10_, NO_2_, O_3_, and SO_2_. These pollutants can influence the disease burden either independently or through combined effects. Even though the GBD 2021 has made certain headway in integrating pollutant data, the combined and interactive impacts of multiple pollutants on the assessment of disease burden are still mostly uninvestigated. (3) The diversity within LRIs suggests varying susceptibilities to PMP, yet data constraints impede an in-depth examination of the relationship between PMP and specific LRI types. (4) Variability in measurement methods, particularly in low-income African nations, might lead to bias and affect the generalizability of the findings (GBD 2021 Causes of Death Collaborators, 2024; GBD 2021 Risk Factors Collaborators, 2024). Given the limitations in PM size-specific data and regional measurement variability, future policies should prioritize standardized monitoring systems to support targeted interventions. Further research is warranted to investigate the associations between additional pollution risk factors and lower respiratory infections. It is essential to develop strategies for clean energy utilization, optimize the distribution of medical resources, and strengthen the enforcement of environmental policies.

## 5 Conclusions

Although there have been substantial global declines in PMP-attributable LRIs mortality and DALYs since 1990, the disparities that remain are striking. Low-SDI regions are still disproportionately burdened, with HAP being a major factor, whereas high-income areas are mainly dominated by APMP.

## Data Availability

The original contributions presented in the study are included in the article/**Supplementary Material**. Further inquiries can be directed to the corresponding authors.

## Supplementary material

Supplementary Figure 1 Global DALYs (Disability-Adjusted Life Years) and age-standardized DALY rates from lower respiratory infections attributable to particulate matter pollution, by five SDI levels, 1990–2021. **(A)** Total particulate matter pollution. **(B)** Household air pollution. **(C)** Ambient particulate matter pollution. Bars represent DALY counts; lines represent age-standardized rates.

Supplementary Figure 2 DALYs from lower respiratory infections attributable to HAP and APMP, by 21 GBD regions and five SDI levels, in 1990 **(A)** and 2021 **(B)**. Proportional distributions are shown for 1990 **(C)** and 2021 **(D)**. Orange represents APMP; green represents HAP.

Supplementary Figure 3 Global distribution of the Estimated Annual Percent Change (EAPC) in age-standardized DALY rates from lower respiratory infections attributable to **(A)** PMP, **(B)** HAP, and **(C)** APMP, 1990–2021.

Supplementary Figure 4 Association between age-standardized DALY rates and Socio-demographic Index for lower respiratory infections due to **(A)** PMP, **(B)** HAP, and **(C)** APMP, across 21 GBD regions, 1990–2021.

Supplementary Figure 5 Association between age-standardized DALY rates and Socio-demographic Index for lower respiratory infections due to **(A)** PMP, **(B)** HAP, and **(C)** APMP, across 204 countries and territories, 1990–2021.

Supplementary Figure 6 Number of deaths (bar chart) and death rates (line chart) of lower respiratory infections attributable to household air pollution, by age group and sex, in 1990 **(A)** and 2021 **(B)**.

Supplementary Figure 7 Number of deaths (bar chart) and death rates (line chart) of lower respiratory infections attributable to ambient particulate matter pollution, by age group and sex, in 1990 **(A)** and 2021 **(B)**.

Supplementary Figure 8 Number of DALYs (bar chart) and DALY rates (line chart) of lower respiratory infections attributable to particulate matter pollution, by age group and sex, in 1990 **(A)** and 2021 **(B)**.

Supplementary Figure 9 Number of DALYs (bar chart) and DALY rates (line chart) of lower respiratory infections attributable to household air pollution, by age group and sex, in 1990 **(A)** and 2021 **(B)**.

Supplementary Figure 10 Number of DALYs (bar chart) and DALY rates (line chart) of lower respiratory infections attributable to ambient particulate matter pollution, by age group and sex, in 1990 **(A)** and 2021 **(B)**.
