# Supplementary figures and images for: Assessing the disease burden of lower respiratory infections attributable to particulate matter pollution: trends from 1990 to 2021 and projections for 2022-2050
Source: Front Cell Infect Microbiol. 2025 Oct 23;15:1660032. doi: 10.3389/fcimb.2025.1660032 (PMC12589054; doi:10.3389/fcimb.2025.1660032)

High SDI   High-middle SDI   Middle SDI   Low-middle SDI   Low SDI

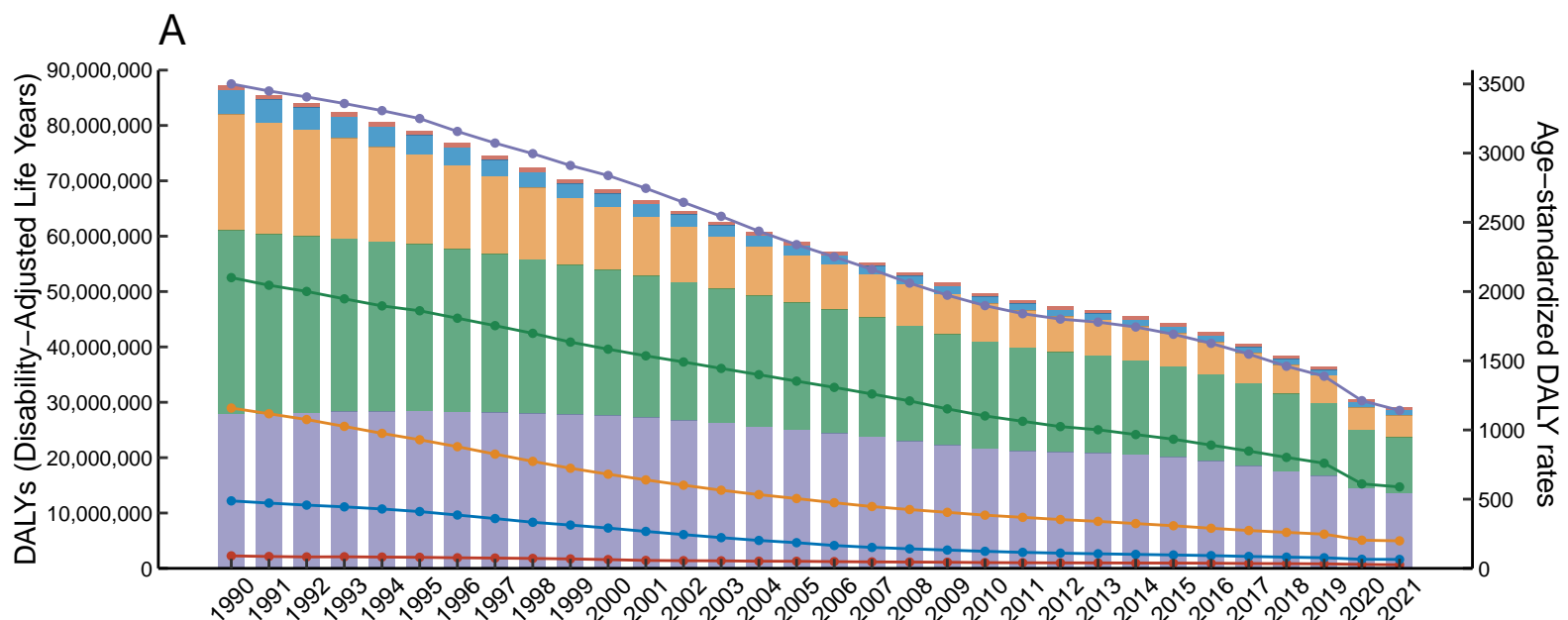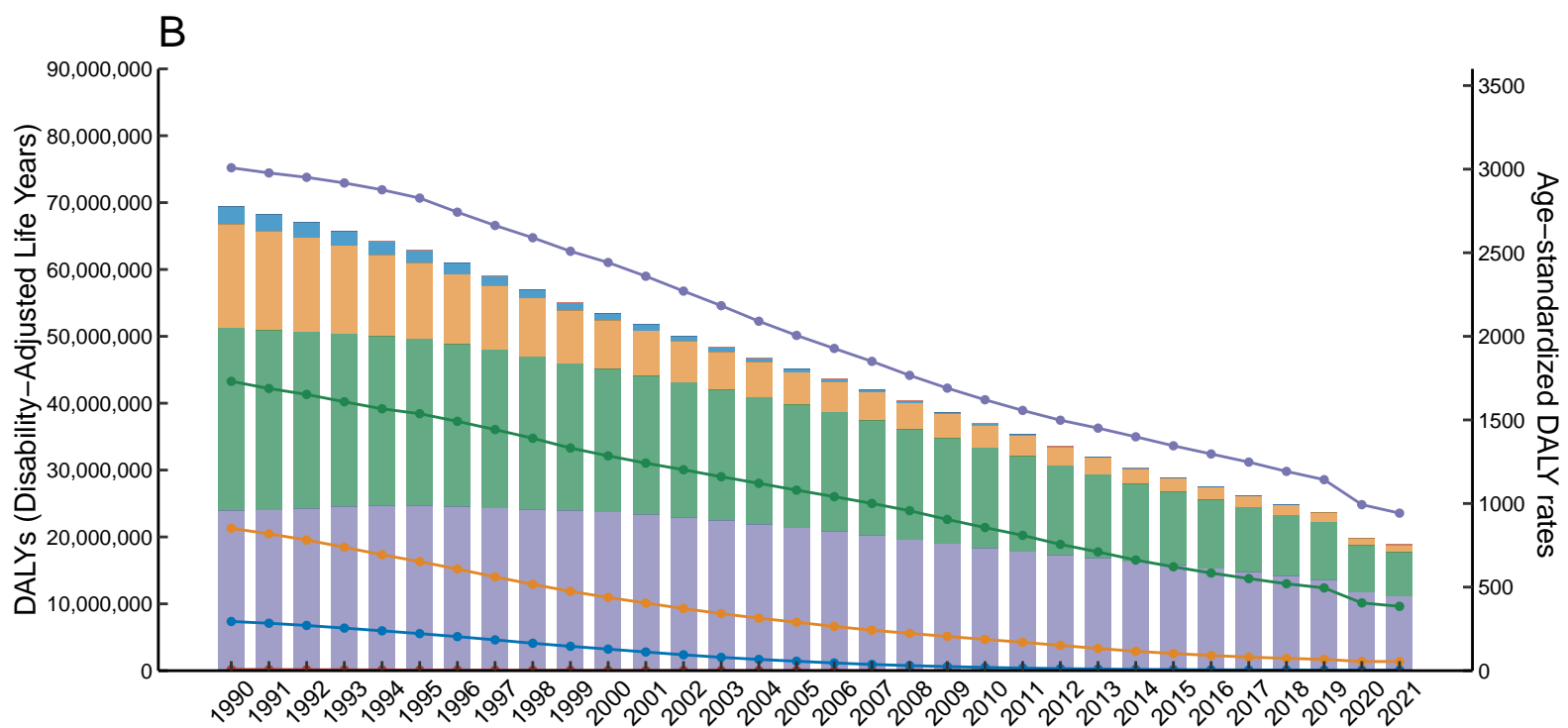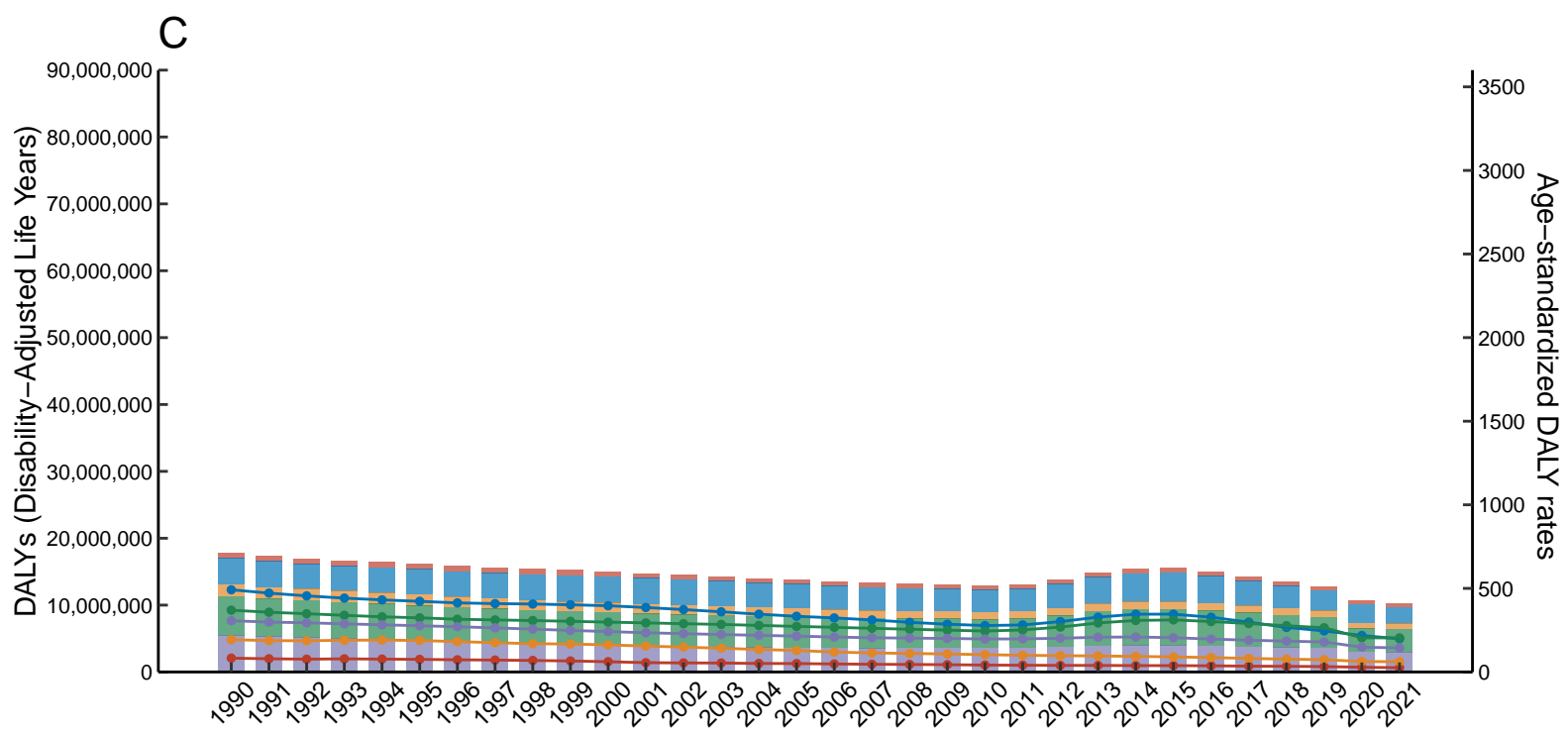

Supplement: Supplementary Figure 1 — Global DALYs (Disability-Adjusted Life Years) and age-standardized DALY rates from lower respiratory infections attributable to particulate matter pollution, by five SDI levels, 1990–2021. (A) Total particulate matter pollution. (B) Household air pollution. (C) Ambient particulate matter pollution. Bars represent DALY counts; lines represent age-standardized rates. [file DataSheet1.pdf]

Ambient particulate matter pollutionHousehold air pollution from solid fuels

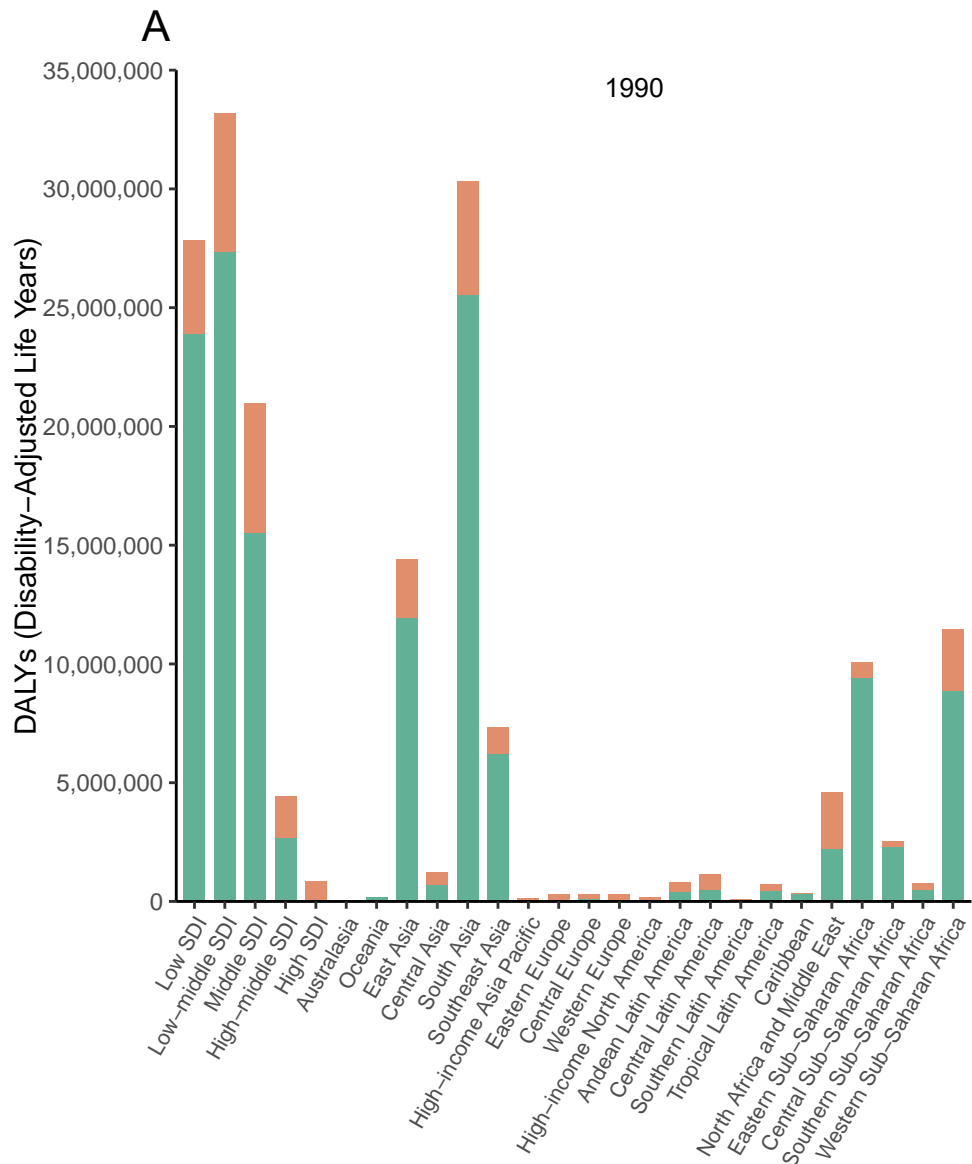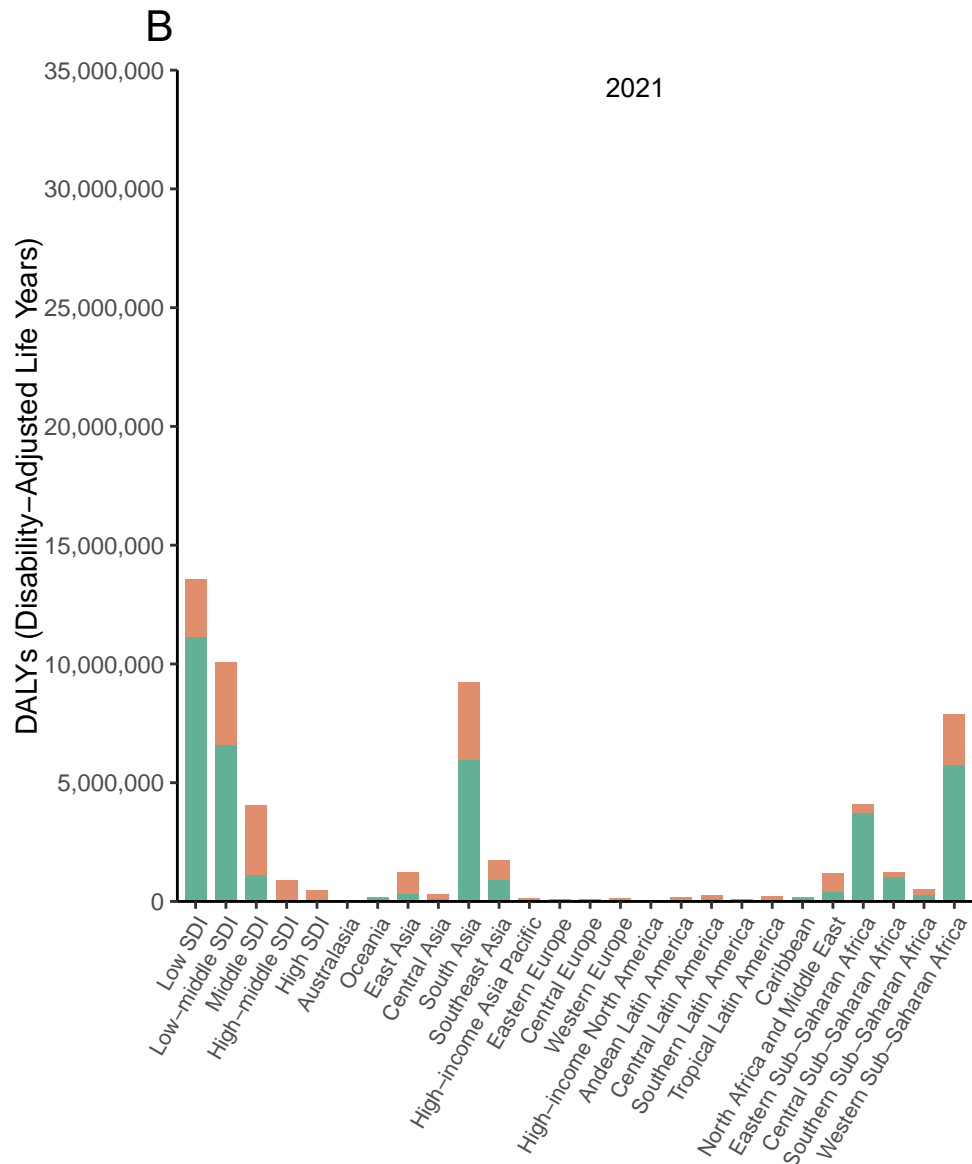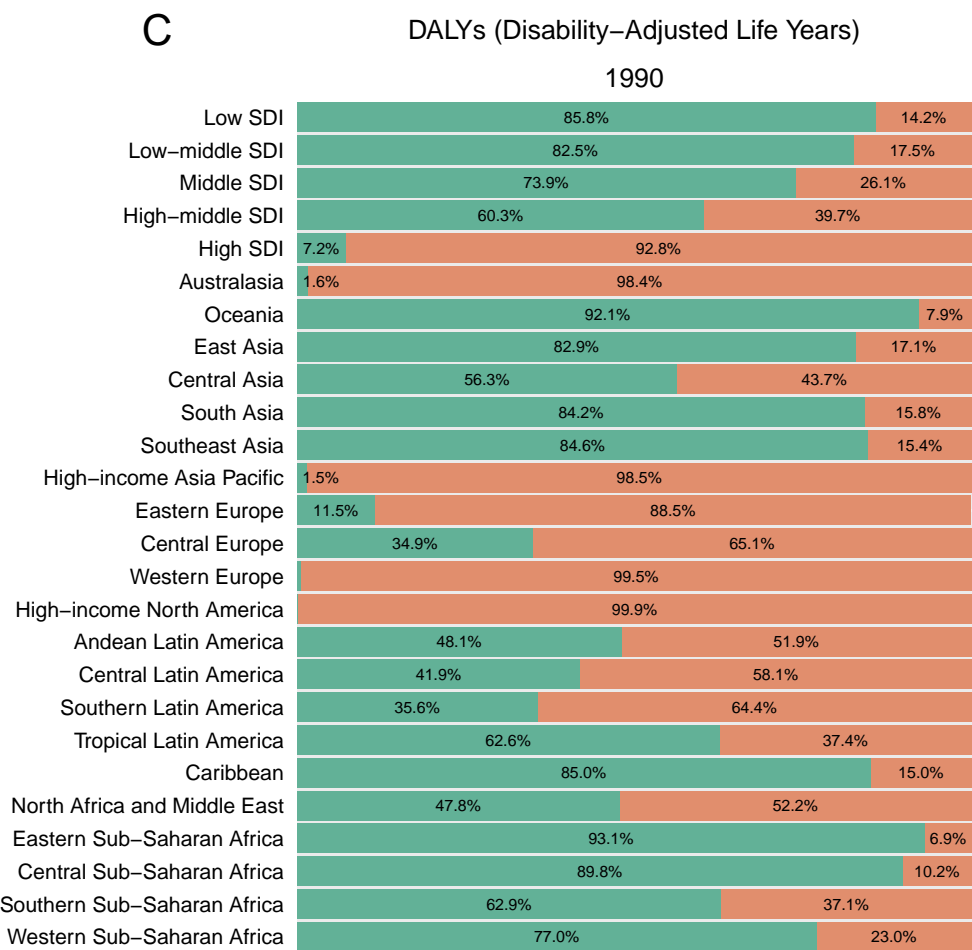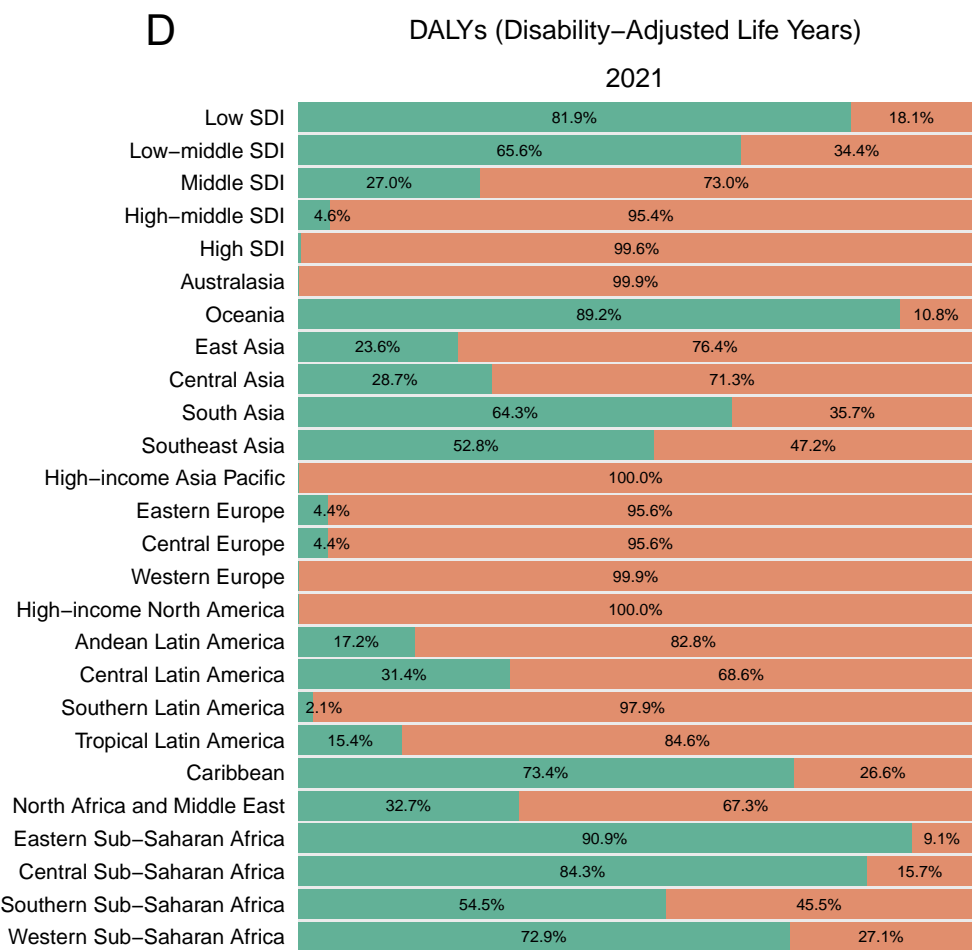

Supplement: Supplementary Figure 2 — DALYs from lower respiratory infections attributable to HAP and APMP, by 21 GBD regions and five SDI levels, in 1990 (A) and 2021 (B). Proportional distributions are shown for 1990 (C) and 2021 (D). Orange represents APMP; green represents HAP. [file DataSheet2.pdf]

A

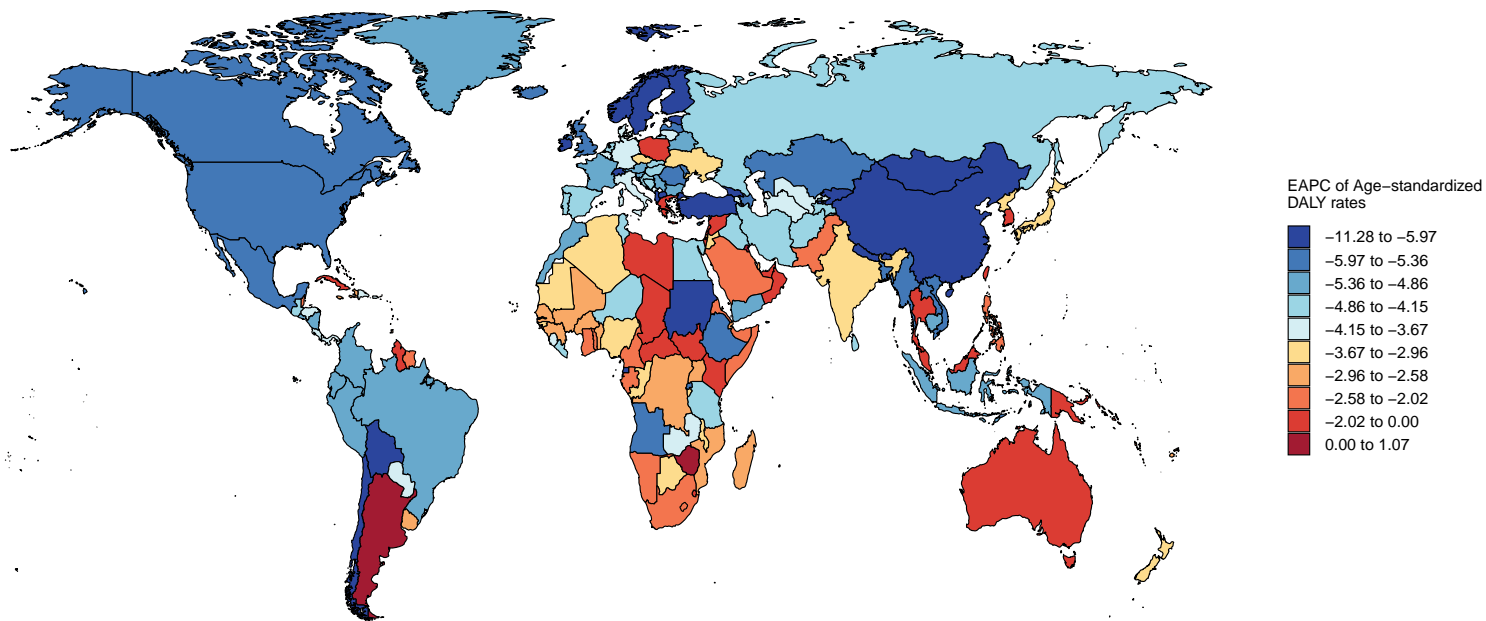

B

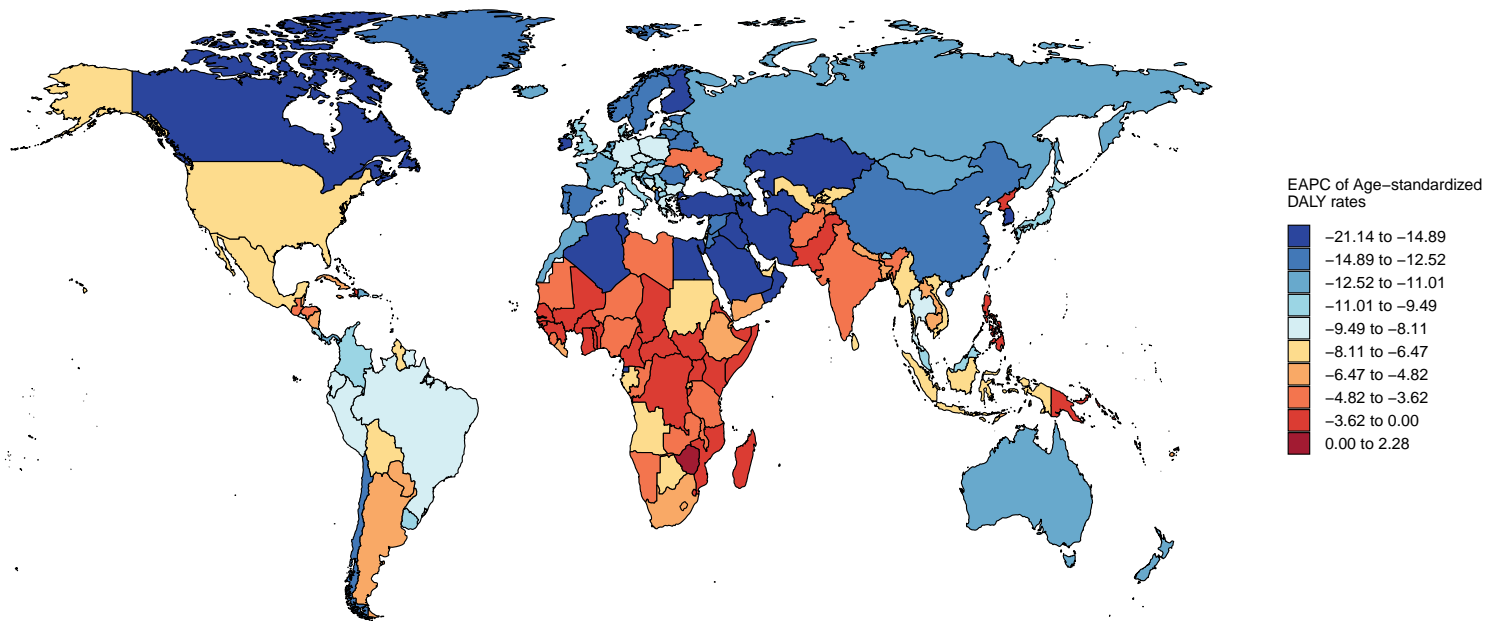

C

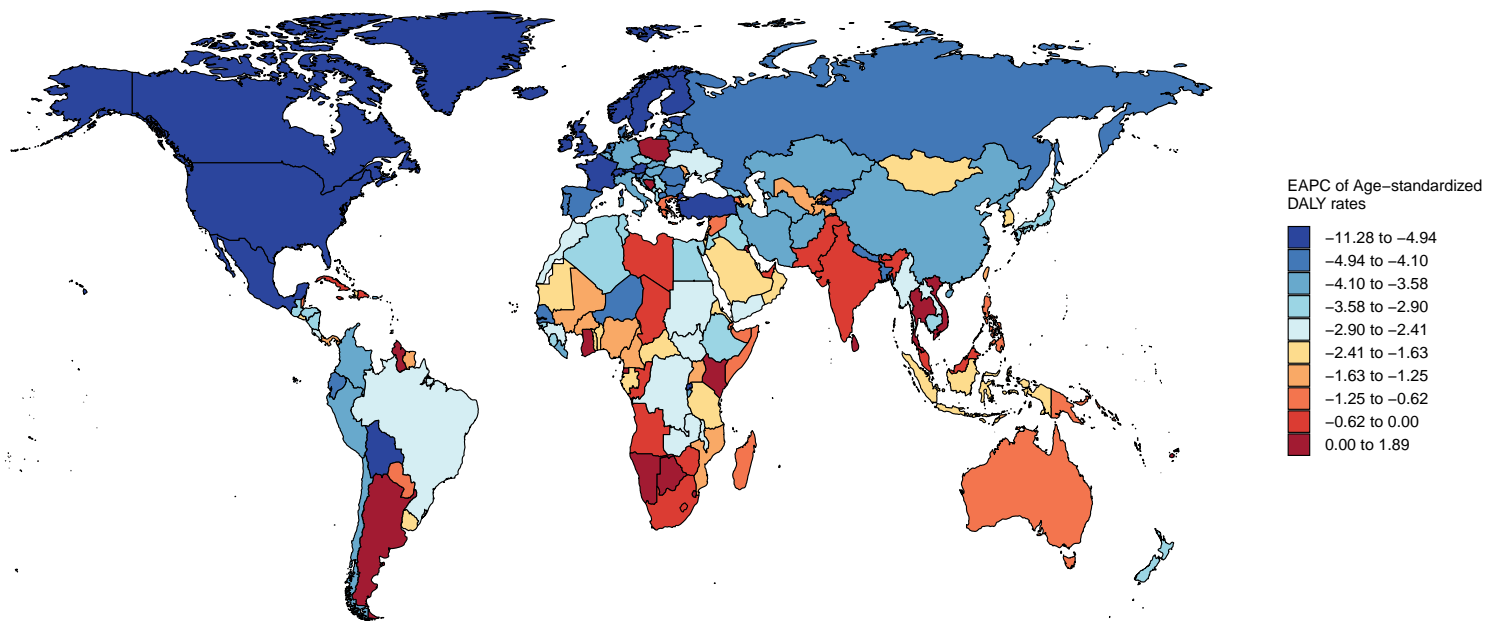

Supplement: Supplementary Figure 3 — Global distribution of the Estimated Annual Percent Change (EAPC) in age-standardized DALY rates from lower respiratory infections attributable to (A) PMP, (B) HAP, and (C) APMP, 1990–2021. [file DataSheet3.pdf]

A

$$r = -0.8576, p < 0.001$$
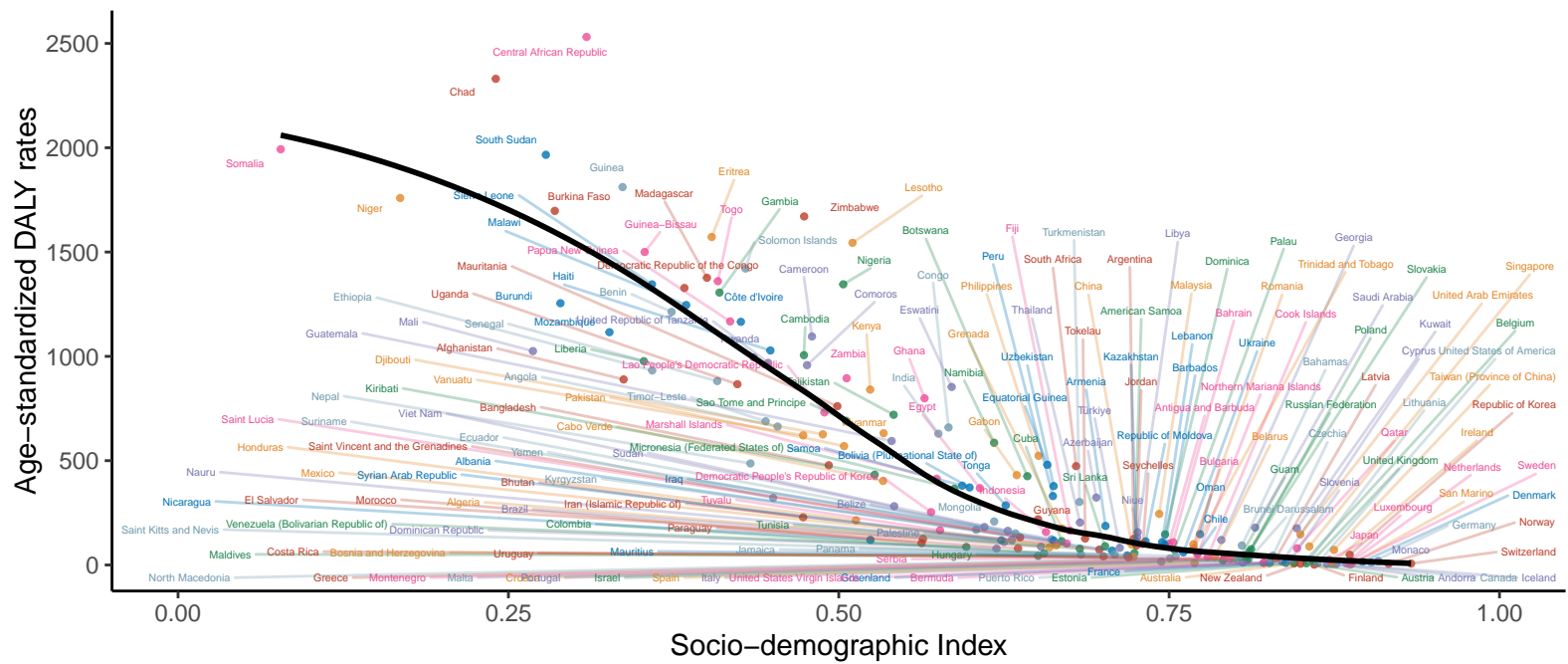

B

$$r = -0.8327, p < 0.001$$
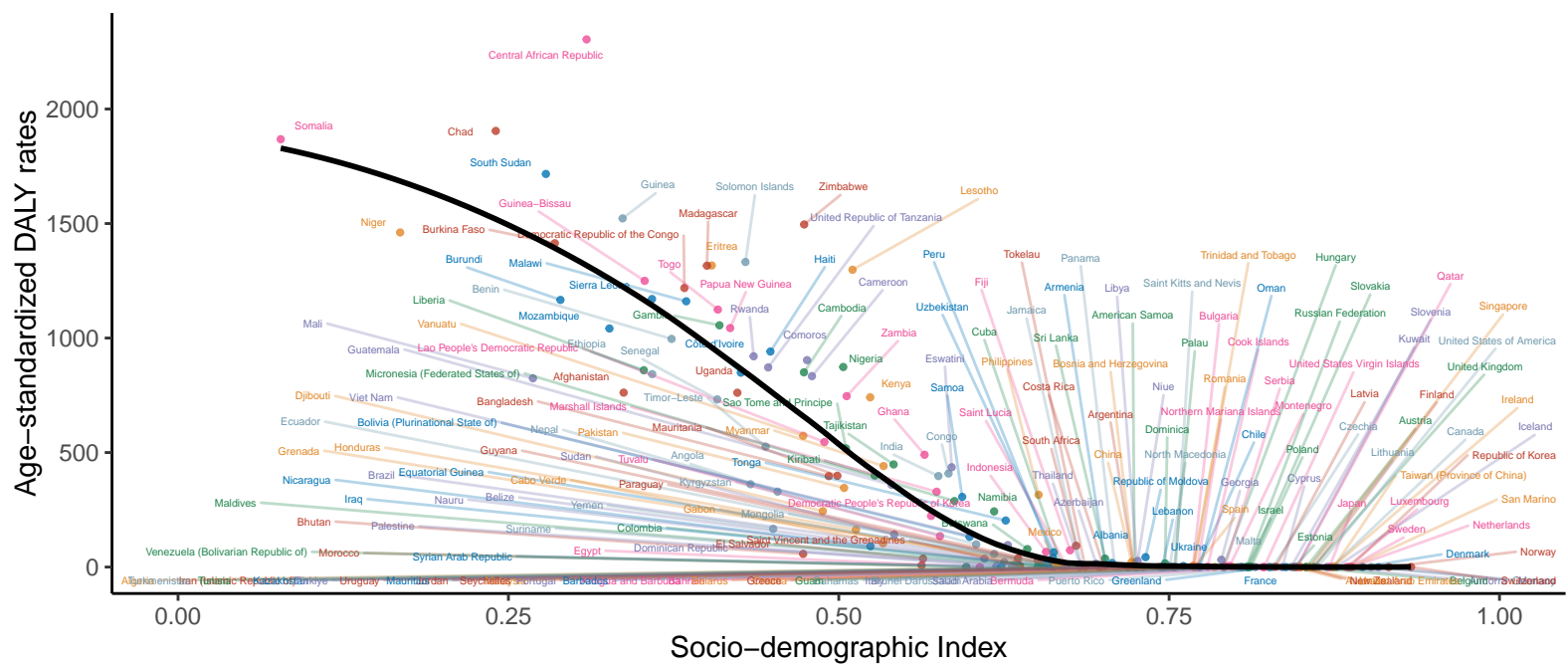

C

$$r = -0.5252, p < 0.001$$
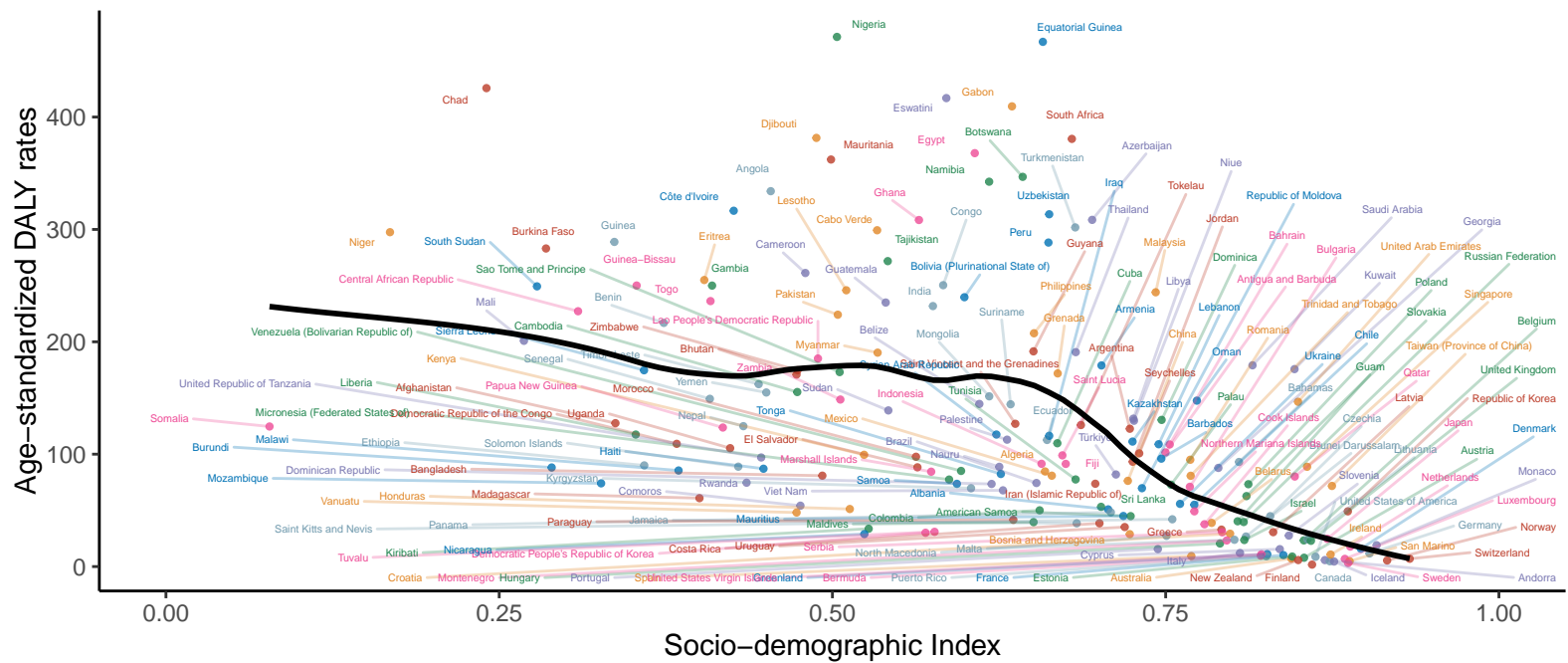

Supplement: Supplementary Figure 5 — Association between age-standardized DALY rates and Socio-demographic Index for lower respiratory infections due to (A) PMP, (B) HAP, and (C) APMP, across 204 countries and territories, 1990–2021. [file DataSheet5.pdf]

A

1990

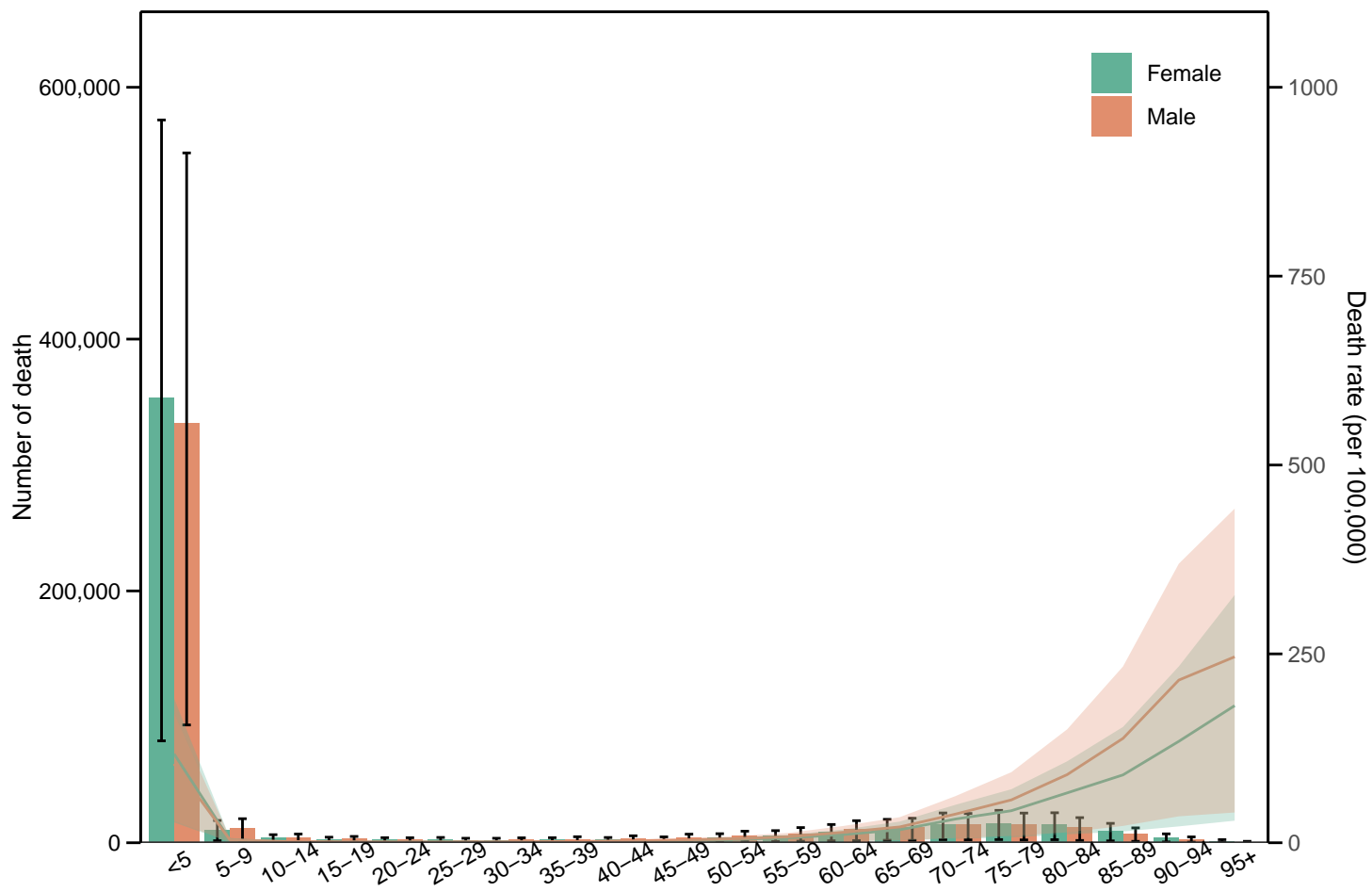

B

2021

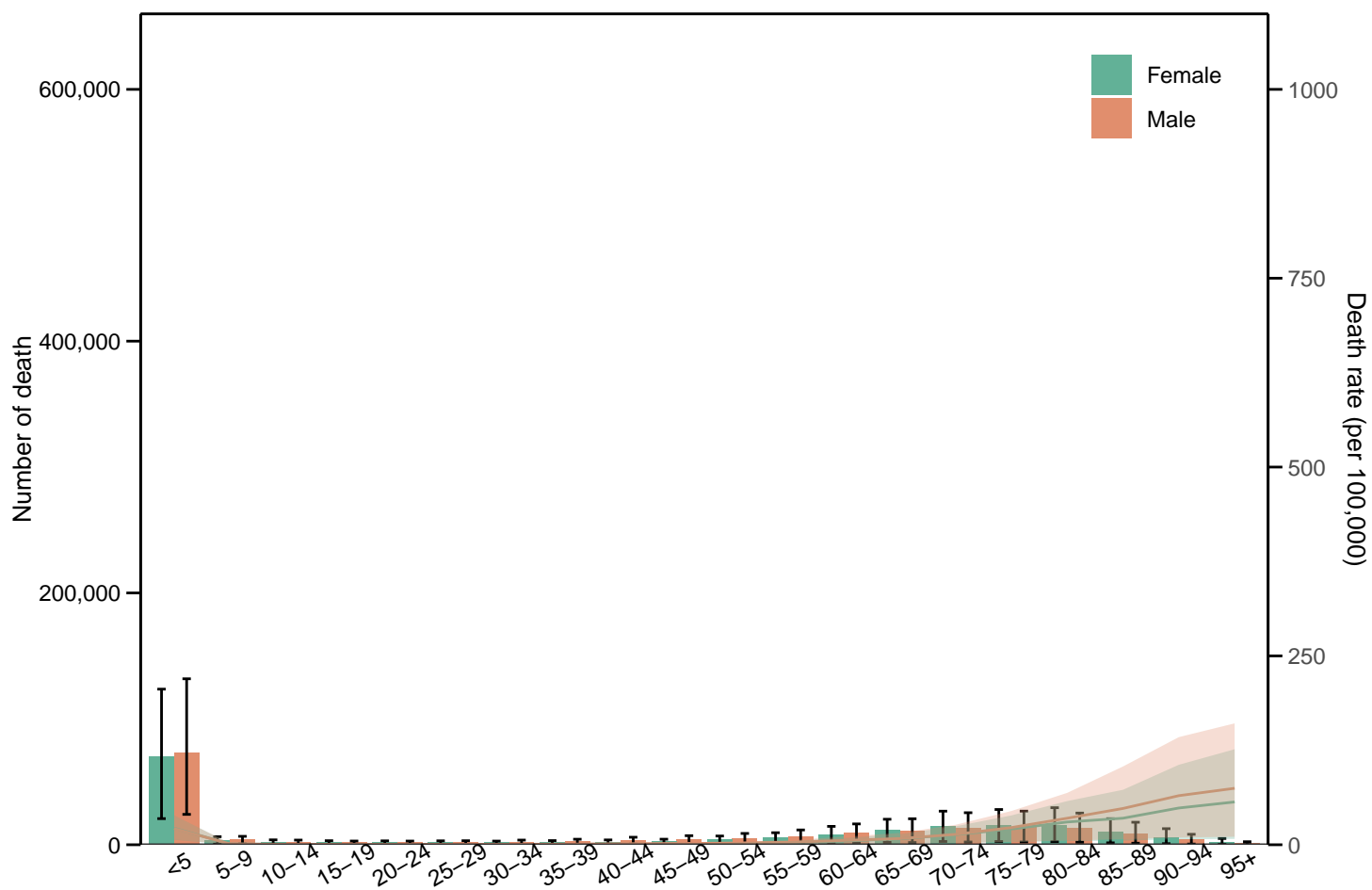

Supplement: Supplementary Figure 6 — Number of deaths (bar chart) and death rates (line chart) of lower respiratory infections attributable to household air pollution, by age group and sex, in 1990 (A) and 2021 (B). [file DataSheet6.pdf]

A

1990

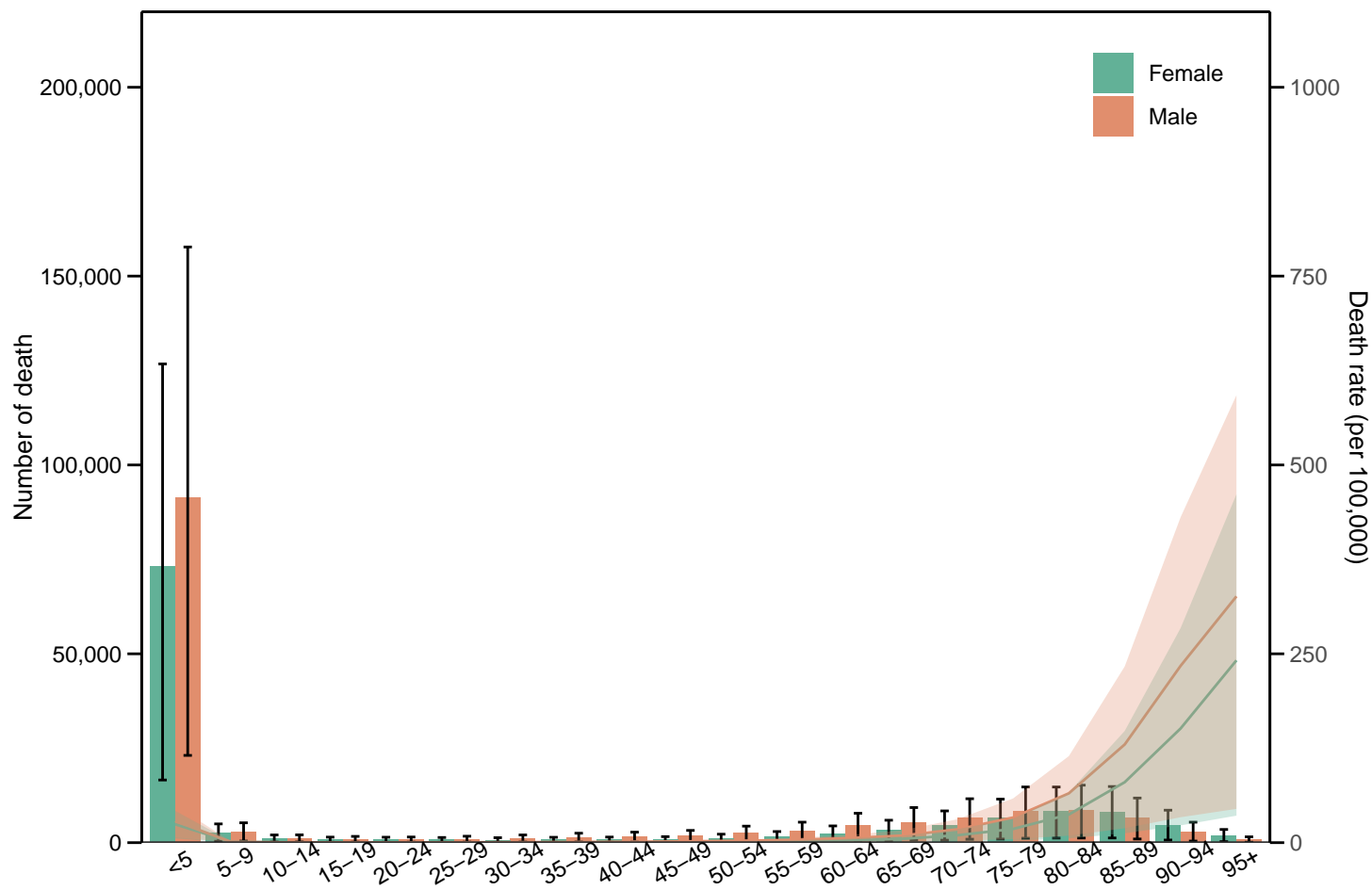

B

2021

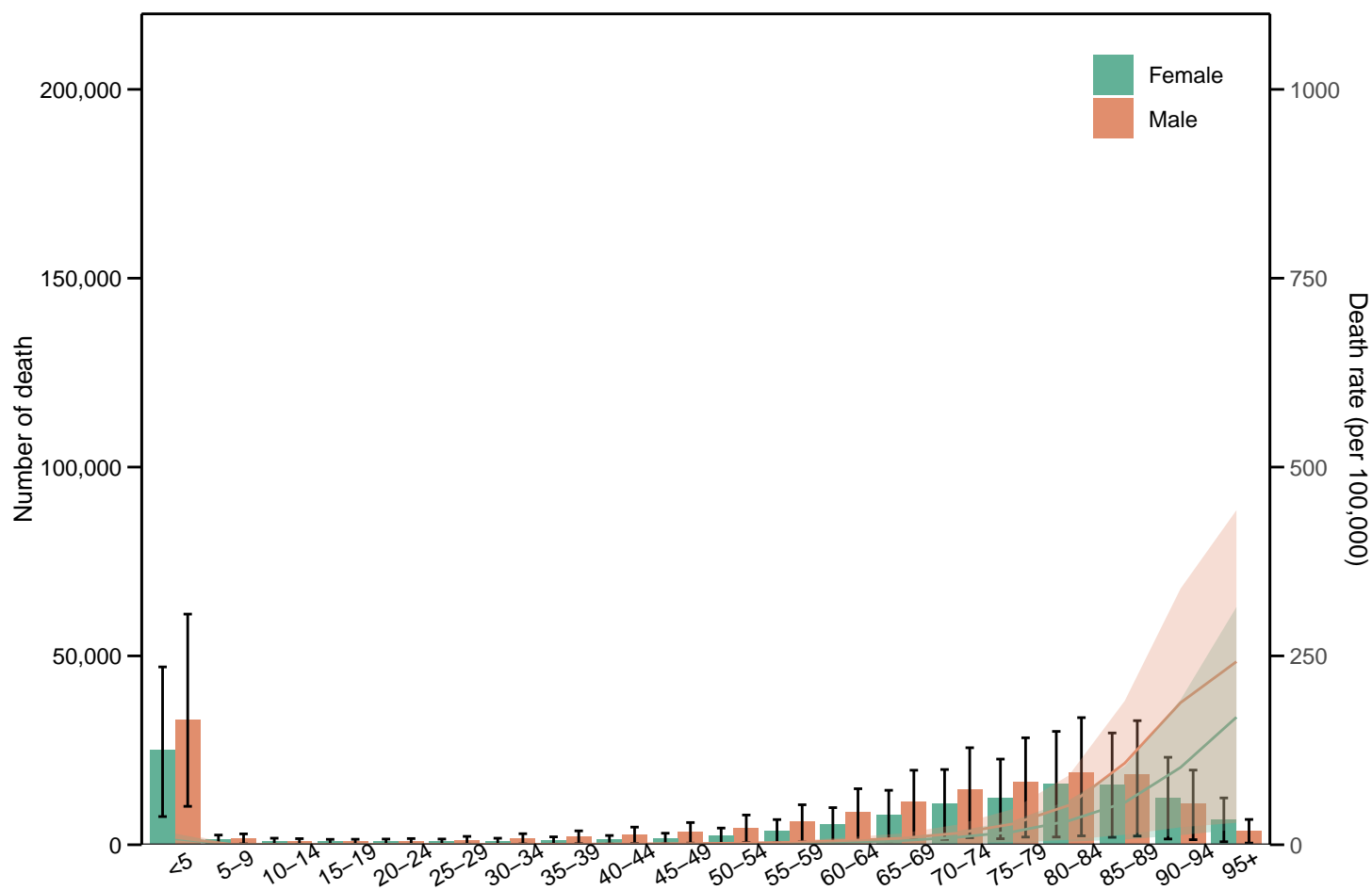

Supplement: Supplementary Figure 7 — Number of deaths (bar chart) and death rates (line chart) of lower respiratory infections attributable to ambient particulate matter pollution, by age group and sex, in 1990 (A) and 2021 (B). [file DataSheet7.pdf]

A

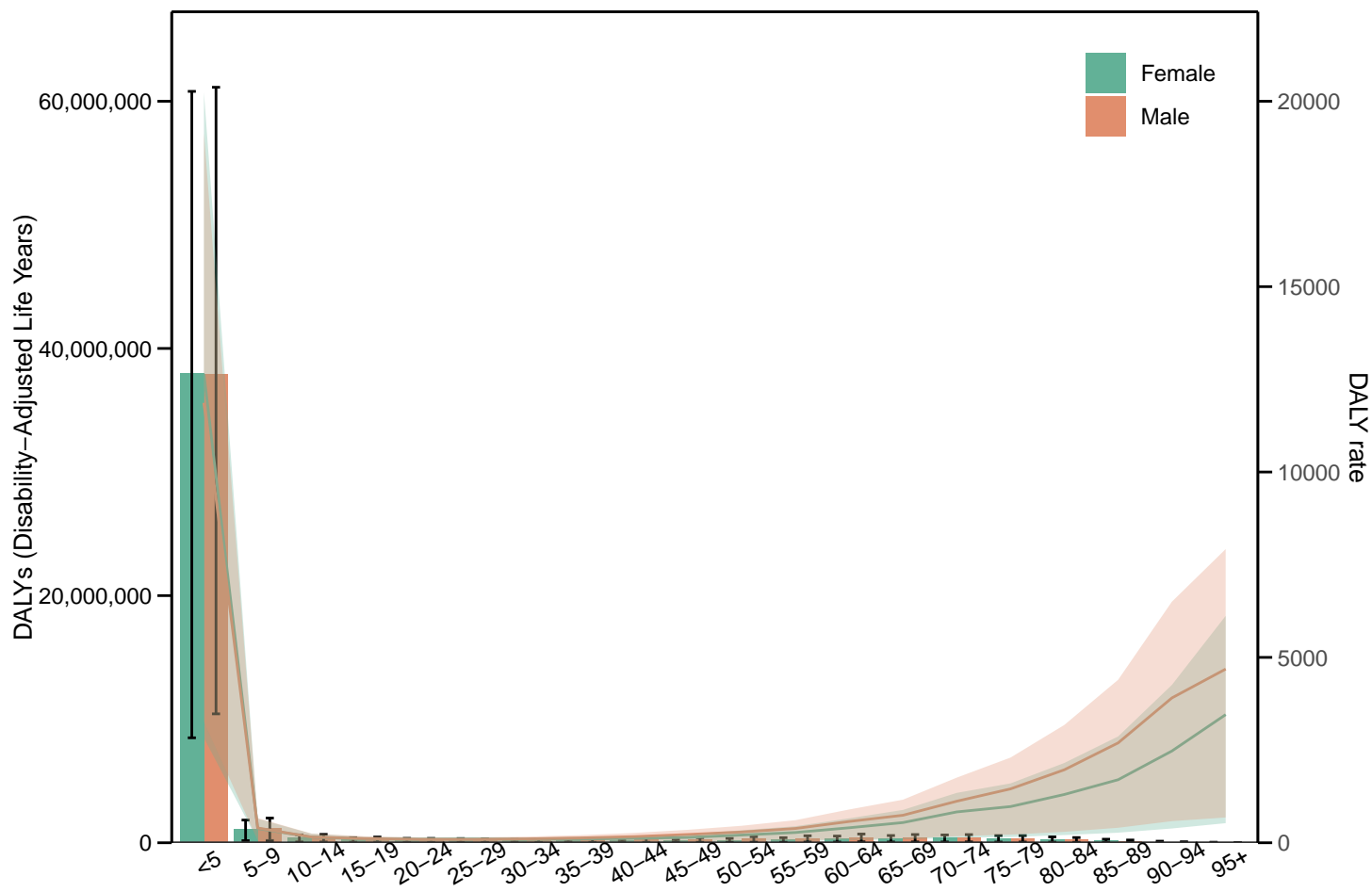

B

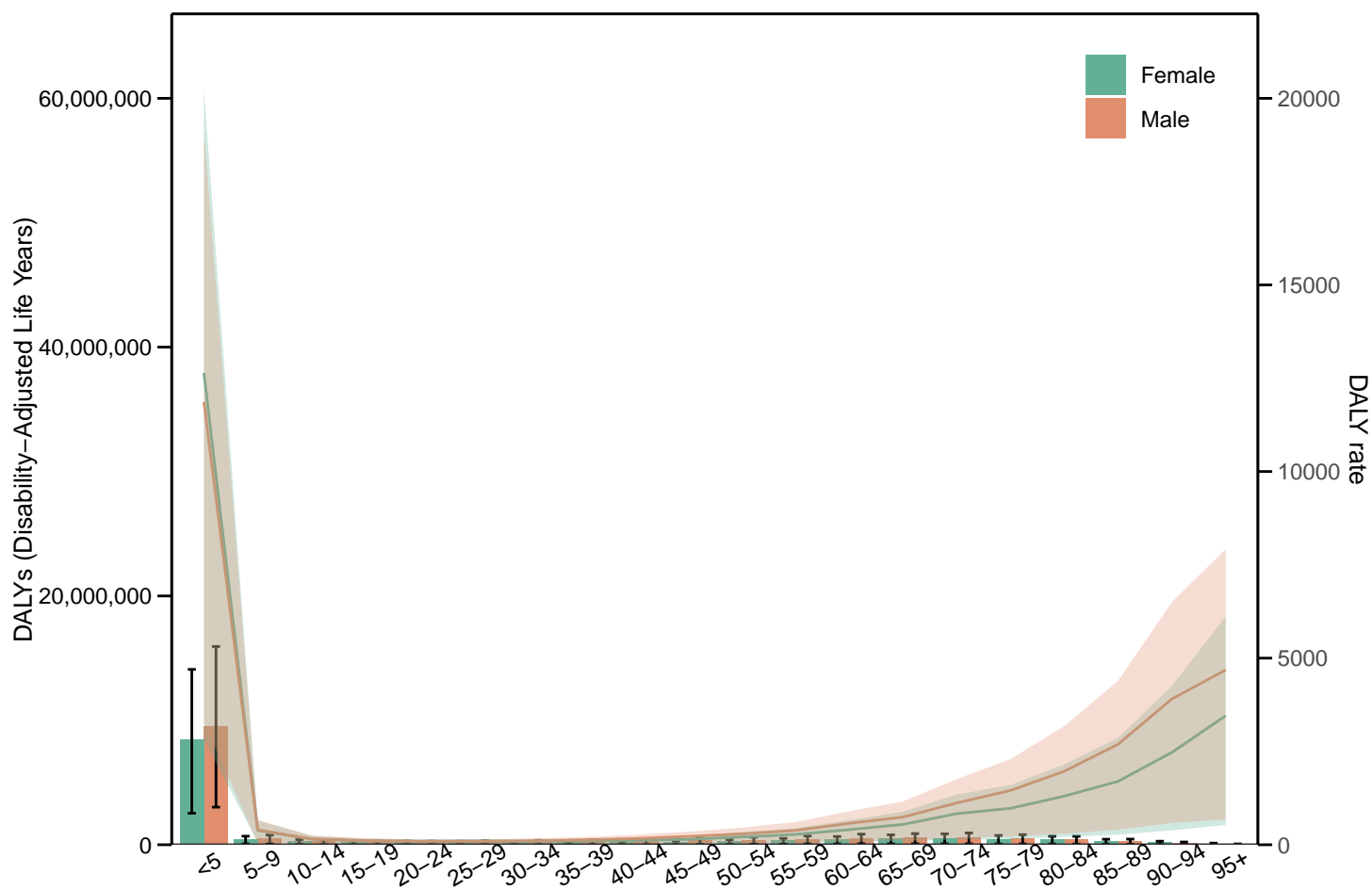

Supplement: Supplementary Figure 8 — Number of DALYs (bar chart) and DALY rates (line chart) of lower respiratory infections attributable to particulate matter pollution, by age group and sex, in 1990 (A) and 2021 (B). [file DataSheet8.pdf]

A

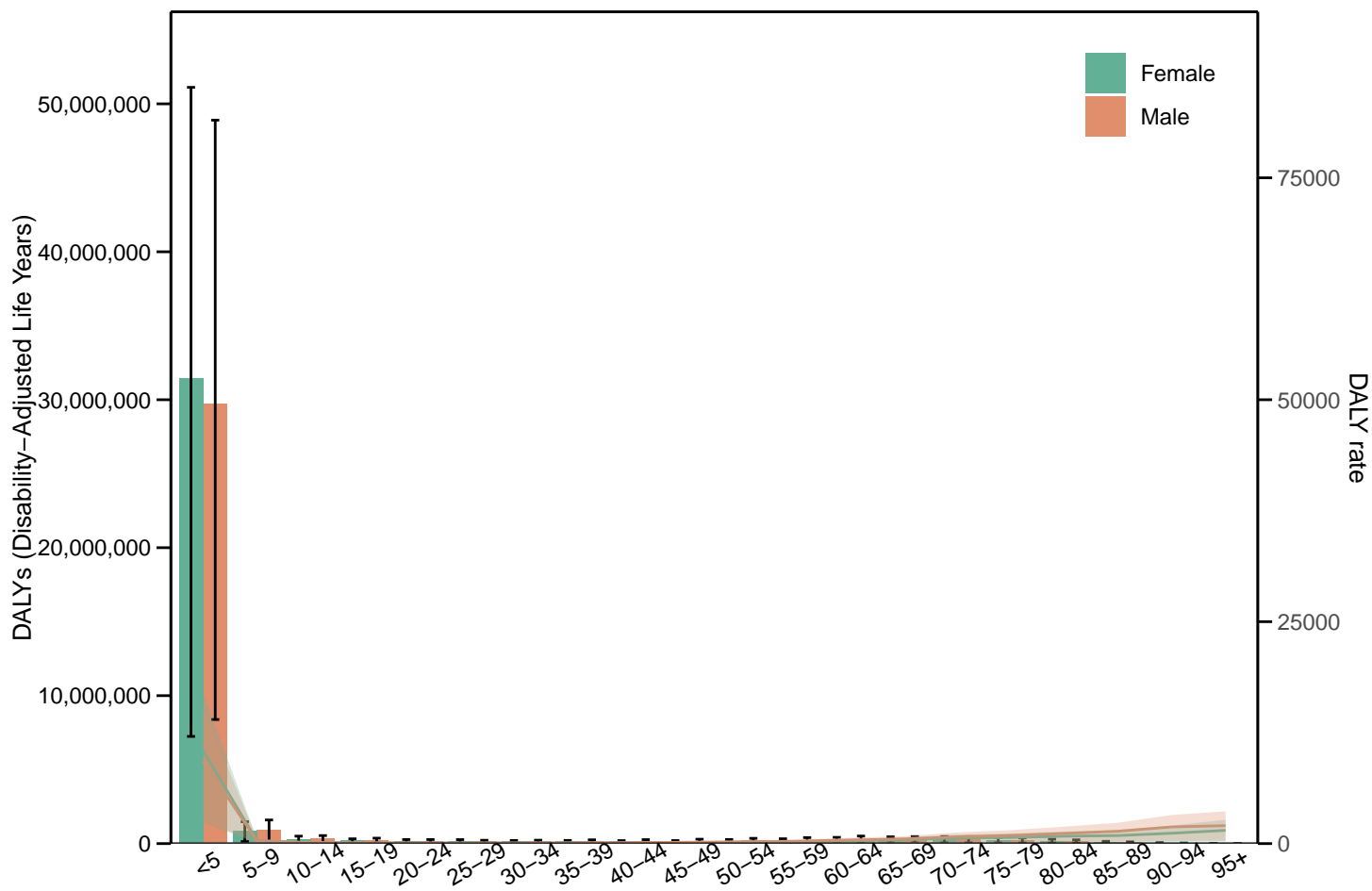

B

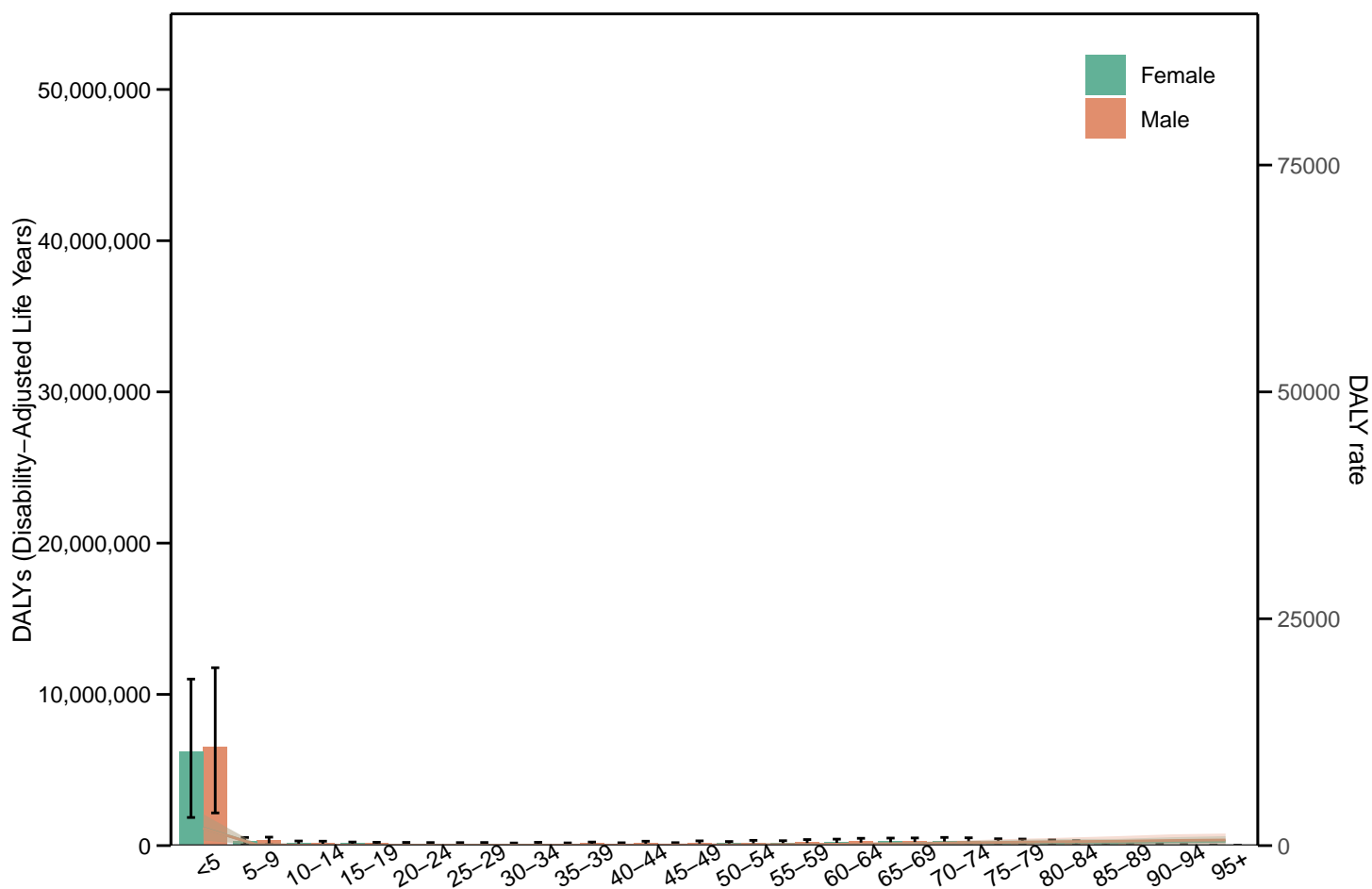

Supplement: Supplementary Figure 9 — Number of DALYs (bar chart) and DALY rates (line chart) of lower respiratory infections attributable to household air pollution, by age group and sex, in 1990 (A) and 2021 (B). [file DataSheet9.pdf]

A

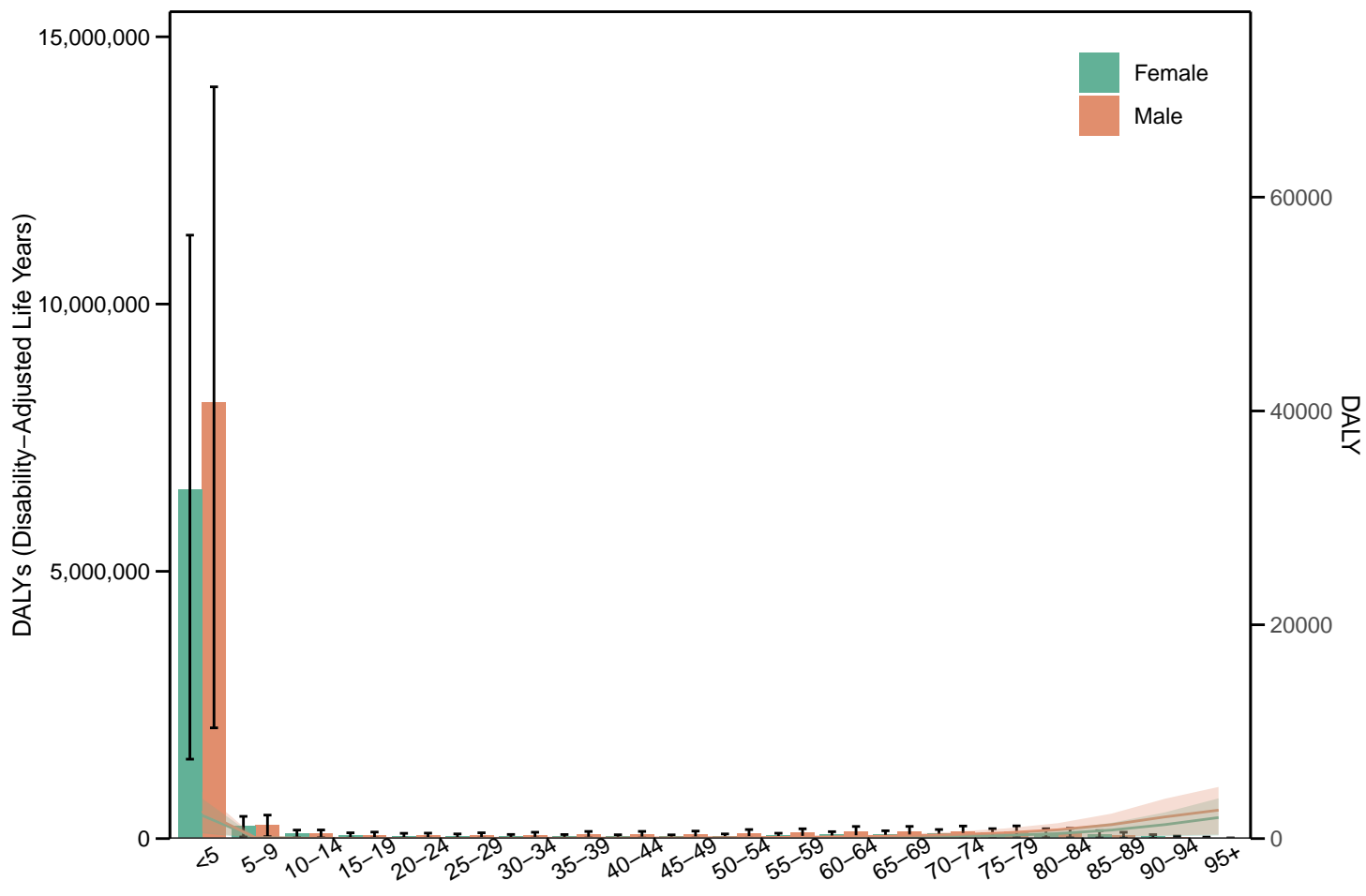

B

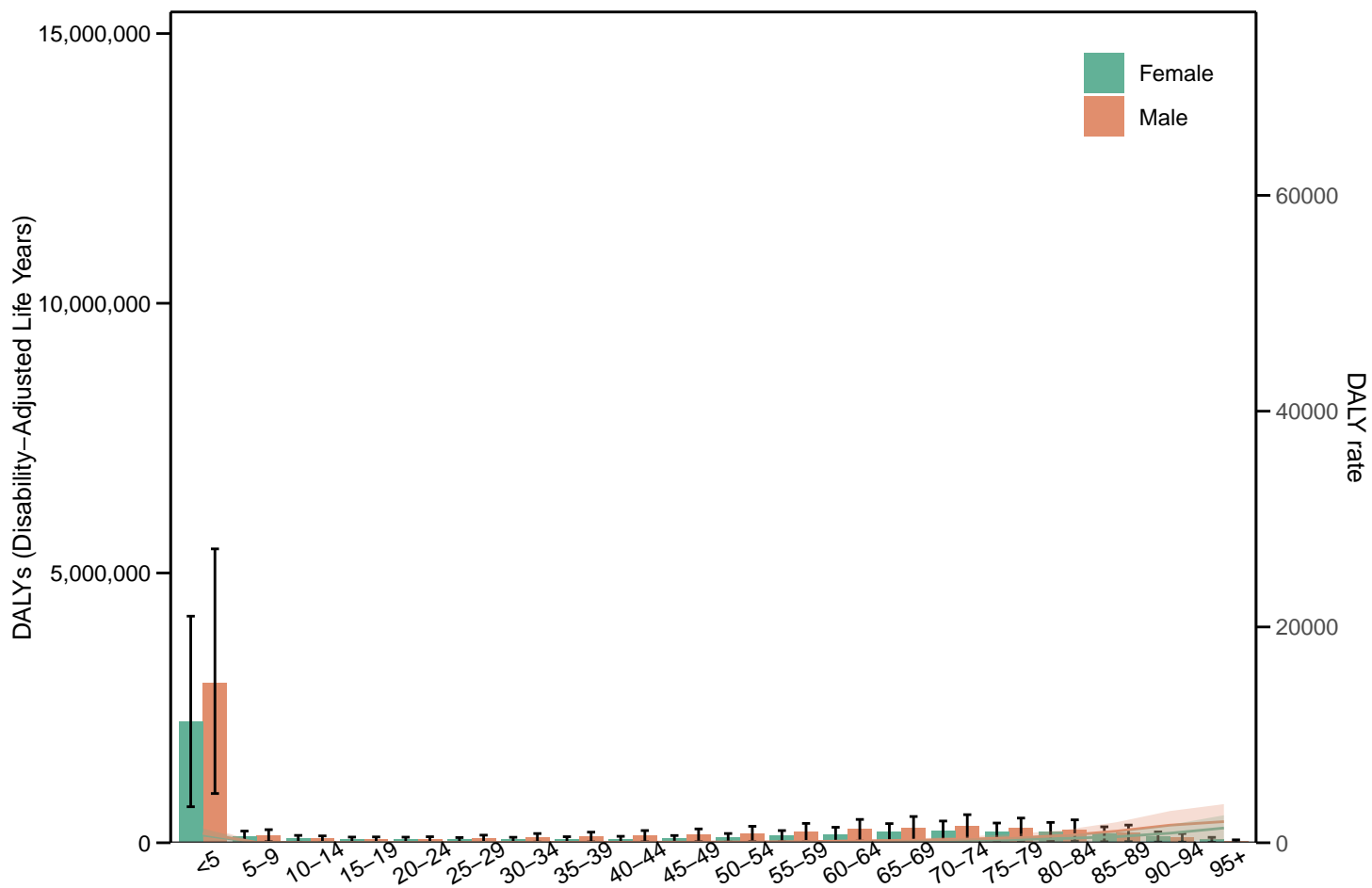

Supplement: Supplementary Figure 10 — Number of DALYs (bar chart) and DALY rates (line chart) of lower respiratory infections attributable to ambient particulate matter pollution, by age group and sex, in 1990 (A) and 2021 (B). [file DataSheet10.pdf]
