# Supplementary material for: Assessing the disease burden of lower respiratory infections attributable to particulate matter pollution: trends from 1990 to 2021 and projections for 2022-2050
Source: Front Cell Infect Microbiol. 2025 Oct 23;15:1660032. doi: 10.3389/fcimb.2025.1660032 (PMC12589054; doi:10.3389/fcimb.2025.1660032)
Supplement: Supplementary Figure 4 — Association between age-standardized DALY rates and Socio-demographic Index for lower respiratory infections due to (A) PMP, (B) HAP, and (C) APMP, across 21 GBD regions, 1990–2021. [file DataSheet4.pdf]

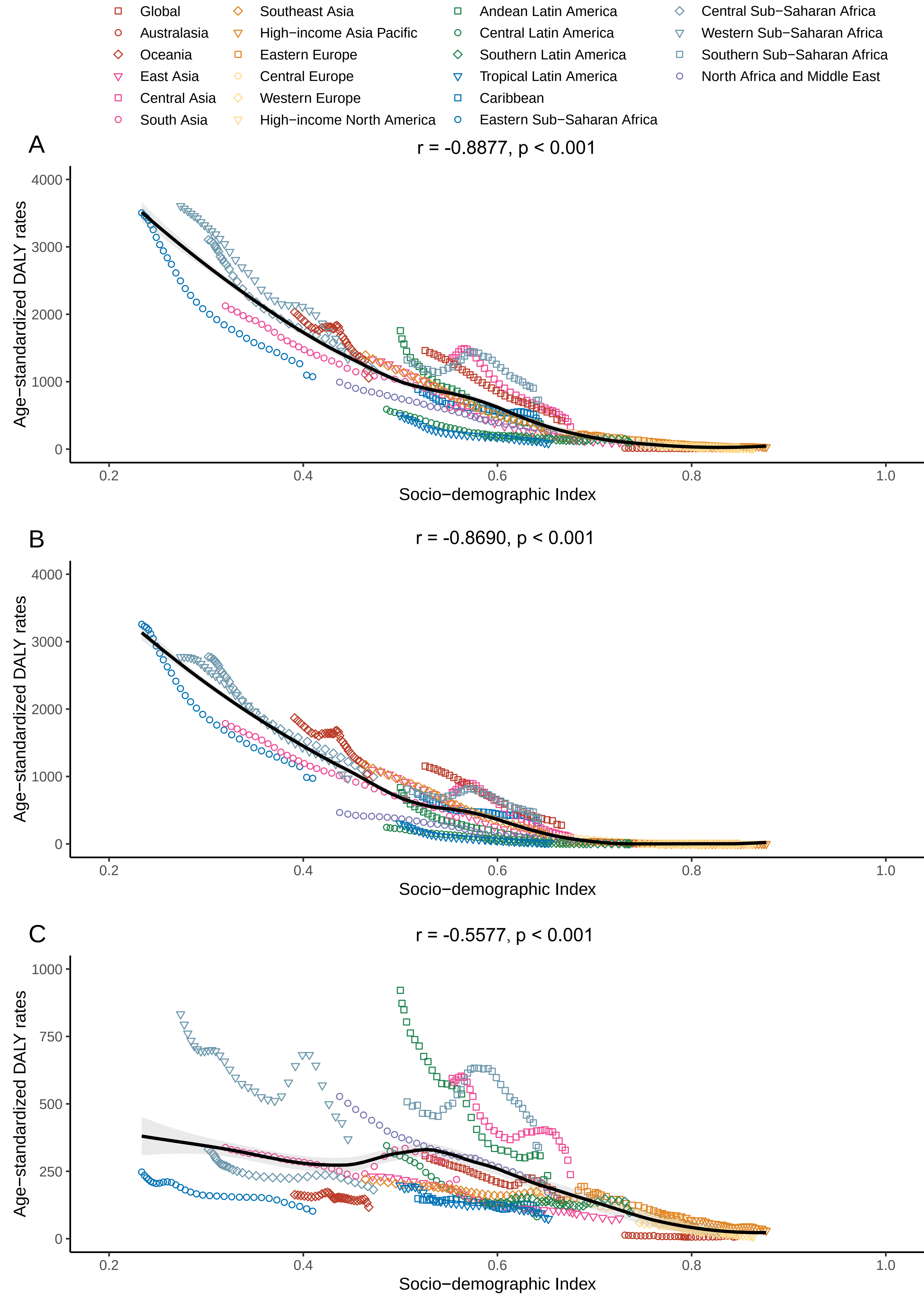

Global
Australasia
Oceania
East Asia
Central Asia
South Asia
Southeast Asia
High−income Asia Pacific
Eastern Europe
Central Europe
Western Europe
High−income North America
Andean Latin America
Central Latin America
Southern Latin America
Tropical Latin America
Caribbean
Eastern Sub−Saharan Africa
Central Sub−Saharan Africa
Western Sub−Saharan Africa
Southern Sub−Saharan Africa
North Africa and Middle East
A
r = -0.8877, p < 0.001
Age−standardized DALY rates
4000
3000
2000
1000
0
0.2
0.4
0.6
0.8
1.0
Socio−demographic Index
B
r = -0.8690, p < 0.001
Age−standardized DALY rates
4000
3000
2000
1000
0
0.2
0.4
0.6
0.8
1.0
Socio−demographic Index
C
r = -0.5577, p < 0.001
Age−standardized DALY rates
1000
750
500
250
0
0.2
0.4
0.6
0.8
1.0
Socio−demographic Index
